# Single‐Cell Dissection Reveals Immune Dysregulation After CD5 or CD7‐Directed Chimeric Antigen Receptor T‐Cell Therapy

**DOI:** 10.1002/advs.202509259

**Published:** 2025-11-25

**Authors:** Yuechen Luo, Haixiao Zhang, Kaiting Tang, Yiming Wang, Huajiang Dong, Wei Qi, Lingling Shan, Yue Tan, Liping Zhao, Jun Shi, Erlie Jiang, Jing Pan, Xiaoming Feng

**Affiliations:** ^1^ State Key Laboratory of Experimental Hematology National Clinical Research Center for Blood Diseases Haihe Laboratory of Cell Ecosystem Institute of Hematology & Blood Diseases Hospital Chinese Academy of Medical Sciences & Peking Union Medical College Tianjin 300020 China; ^2^ Tianjin Institutes of Health Science Tianjin 301600 China; ^3^ Novogene Co., Ltd. Beijing 100015 China; ^4^ Logistics University of Chinese People's Armed Police Forces Tianjin 300309 China; ^5^ State Key Laboratory of Experimental Hematology Department of Hemato‐oncology and Immunotherapy Beijing GoBroad Hospital Beijing 102206 China; ^6^ Central laboratory Fujian Medical Union Hospital Fuzhou 350001 China

**Keywords:** CAR T cells, immunodeficiency, single‐cell sequencing

## Abstract

CD5‐ and CD7‐directed chimeric antigen receptor T‐cell (5CAR and 7CAR) therapies for T‐cell malignancies carry the risk of life‐threatening infection. Although depletion of target‐positive lymphocytes is expected, the contribution of residual cell dysfunction to infection risk remains unclear. This work uses single‐cell sequencing to investigate immune dysregulation after 5CAR or 7CAR therapy in patients with T‐cell acute lymphoblastic leukemia. 5CAR induces marked T‐cell exhaustion linked to CD5 loss and B lymphocyte‐induced maturation protein 1 upregulation. This is accompanied by reduced frequency and diversity of Epstein‐Barr virus (EBV)‐associated T‐cell receptors, potentially contributing to the high incidence of severe EBV infection. 5CAR therapy also impairs B‐cell function and diversity while enhancing natural killer cell function and monocyte activation. In contrast, 7CAR reduces the frequency and diversity of multiple pathogen‐associated T‐cell receptors, but causes less T‐cell exhaustion. 7CAR also substantially impairs innate immunity by decreasing monocyte activation and eliminating dendritic cells, which may contribute to the high risk of infection. Thus, unlike CD19 and CD22 CAR therapy, which primarily affects B cells, 5CAR and 7CAR therapies result in broad dysregulation across multiple immune cell types, providing a basis for infection prevention and safer CAR‐T therapy.

## Graphical abstract

Single‐cell RNA/TCR/BCR sequencing reveals that 5CAR therapy in T‐ALL induces T‐cell exhaustion, reduces EBV‐associated TCRs, lowers TCR/BCR diversity, and increases NK/DC/monocyte activation and function. In contrast, 7CAR therapy reduces multiple pathogen‐associated TCRs, enhances NK cell activation and function, decreases monocyte activation, and eliminates DCs. These findings suggest a potential approach for preventing infections after CAR‐T therapy.

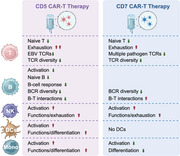

## 1 Introduction

T‐cell acute lymphoblastic leukemia (T‐ALL) is typically characterized by the presence of cytoplasmic or cell‐surface CD3. It originates from clones at various thymic stages and is classified into pro‐T, pre‐T/immature, cortical‐T, and mature T subtypes.^[^ ^1^ ^]^ Compared with B‐cell acute lymphoblastic leukemia (B‐ALL), T‐ALL is more prone to extramedullary relapse and exhibits a poorer response to chemotherapy,^[^ ^2^ ^]^ with limited treatment options for relapsed or refractory T‐ALL (r/r T‐ALL).^[^ ^2^, ^3^ ^]^ Although chimeric antigen receptor T‐cell (CAR‐T) therapy has revolutionized the treatment of B‐cell malignancies,^[^ ^4^ ^]^ its application in T‐ALL is limited by challenges in manufacturing autologous products, self‐targeting among CAR‐T cells, and toxicity to normal hematopoietic cells.^[^ ^5^, ^6^, ^7^ ^]^

Strategies have been proposed in clinical trials to improve CAR‐T cell therapy for T‐ALL, including the investigation of various target antigens.^[^ ^5^, ^7^, ^8^, ^9^, ^10^ ^]^ CD7 is expressed on most human T cells, natural killer (NK) cells, and T‐ALL cells and participates in signal transduction in both T and NK cells.^[^ ^11^, ^12^, ^13^ ^]^ CD5 is found on the surface of T cells and a subset of B cells and regulates the activation of T and B cells and immune tolerance. CD5 is expressed on most T‐ALL cells and is associated with a favorable prognosis in childhood T‐ALL.^[^ ^14^, ^15^ ^]^ Our team has conducted trials of HLA‐matched donor‐derived CD7 and CD5 CAR‐T (7CAR and 5CAR) cells in patients with r/r T‐ALL and achieved high complete remission rates. However, these therapies are associated with severe viral, fungal, and bacterial infections. The rate of severe infection (grade ≥3) was 25% (with 20% related mortality) after 7CAR therapy, and 43.8% (with 31.3% related mortality) after 5CAR therapy.^[^ ^7^, ^8^, ^16^ ^]^ Severe infection post CD19 and CD22 CAR‐T cell (19+22CAR) therapy was less common, with a rate of 7.4% and no related death in our previous study.^[^ ^17^ ^]^ Severe infections have also been reported following 7CAR or 5CAR therapy for T‐ALL in other studies.^[^ ^18^, ^19^, ^20^ ^]^ Although reduced normal lymphocytes counts may contribute to a high infection risk,^[^ ^7^ ^]^ the functionality of the remaining target antigen‐negative cells remains unclear.

A single‐cell RNA sequencing (scRNA‐seq) study showed that CD7‐negative T cells retained near‐normal function, whereas reduced monocytes may be associated with severe infections after 7CAR therapy.^[^ ^21^ ^]^ However, immune system dysregulation following 7CAR therapy remains largely unexplored, and immune alterations following 5CAR therapy have not been characterized. Here, we employed single‐cell sequencing to analyze the immune cells of patients who received 5CAR or 7CAR therapy. For reference, we also included patients with B‐ALL treated with 19+22CAR cells, which primarily deplete B cells. This design allowed us to determine whether the immune alterations following 5CAR or 7CAR T‐cell therapies were specific to T‐cell depletion or extended to other immune subsets. The clinical course of CD19 CAR‐T cell therapy is well characterized and is associated with manageable infections. This provides a more relevant clinical benchmark than the use of healthy donors (HDs) alone. Our study revealed broad immunological disturbances following the infusion of 5CAR and 7CAR T cells, providing a foundation for addressing critical clinical challenges associated with immune dysregulation induced by these approaches.

## 2 Results

### 2.1 Integrated Analysis of Single‐Cell Sequencing Data

**Figure 1:**
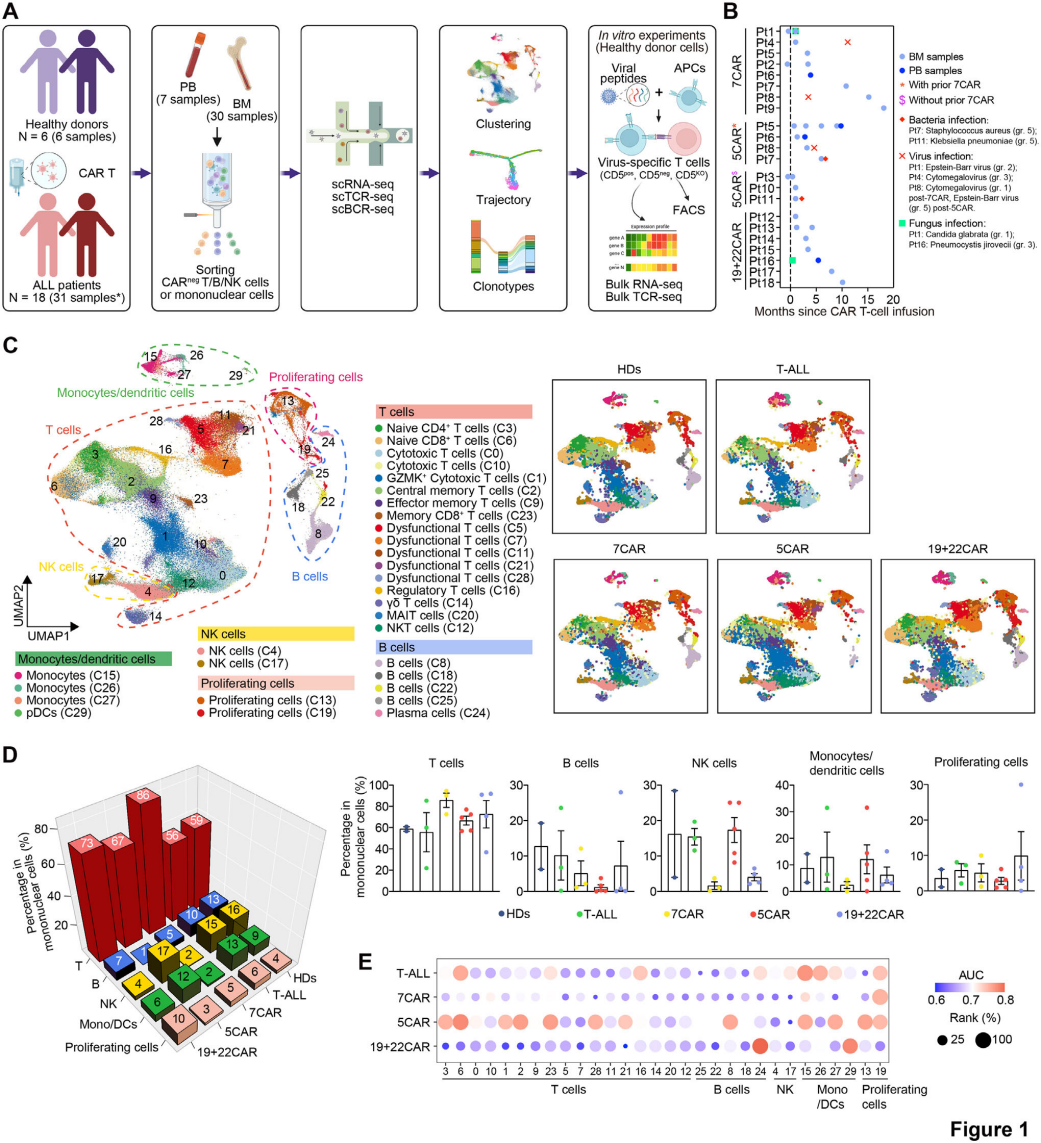
Integrated analysis of single‐cell sequencing data. A) Study overview. *These 18 patients include seven in the 5CAR group, eight in the 7CAR group, seven in the 19+22CAR group, and three in the T‐ALL group. Four patients (Pt 5, 6, 7 and 8) received 7CAR prior to 5CAR, and three T‐ALL patients subsequently received 7CAR (Pt 1 and 2) and 5CAR (Pt 3). Therefore, samples from seven patients were included in two groups simultaneously. B) Timeline of patient sample collection and infection onset. C) UMAP visualization of all captured cells (left) and cells in each group (right). D) 3D bar plot showing the proportion of each cell type, averaged from all donors/patients in each group (left), alongside the individual data for each sample (right). Only samples containing mononuclear cells were used. One sample (Sample 8) from the 19+22CAR group, collected 1.2 months post‐infusion, showed a relatively high B cell proportion, despite flow cytometry indicating B cell aplasia (0.19% of lymphocytes in the peripheral blood). Data are presented as means ± SEMs, n = 2 in HDs, n = 3 in T‐ALL, n = 3 in 7CAR, n = 5 in 5CAR, n = 4 in 19+22CAR group. E) Prioritization of the most affected cell types in different conditions relative to HDs condition by ranking Augur Cell Type Prioritization scores. Samples containing either mononuclear cells or lymphocytes (T, B, and NK cells) were used. APC, antigen‐presenting cells; BCR, B‐cell receptor; BM, bone marrow; CAR, chimeric antigen receptor; FACS, Fluorescence‐Activated Cell Sorting; gr., grade; PB, peripheral blood; Pt, patient; TCR, T‐cell receptor.

To characterize the immune cell landscape, we performed scRNA‐seq on sorted non‐tumor, non‐CAR mononuclear cells and lymphocytes (T, B, and NK cells) from 37 samples. These samples included seven patients with T‐ALL post 5CAR therapy, eight patients with T‐ALL post 7CAR therapy, seven patients with B‐ALL post 19+22CAR therapy, which was included as a comparator that predominantly affects B cells, three patients with T‐ALL before lymphodepletion and CAR therapy (T‐ALL, two and one subsequently received 7CAR and 5CAR therapy, respectively), and six HDs (**Figure** **1**A,B, Figure S1A and Table S1, Supporting Information). Four patients with T‐ALL who relapsed after the initial 7CAR therapy underwent subsequent 5CAR treatment and were included in both the 7CAR and 5CAR groups. Since 38% of the patients underwent stem cell transplantation within 2 months of 5CAR therapy,^[^ ^7^ ^]^ only early stage samples were available for three of the seven patients post 5CAR therapy. To minimize sampling time‐point biases, we analyzed all available samples but focused primarily on those collected within 2 months post‐infusion for many analyses. Thirty samples were obtained from the bone marrow (BM) and seven from the peripheral blood (PB). Thirty‐seven and 25 samples underwent single‐cell T‐cell receptor sequencing (scTCR‐seq) and single‐cell B‐cell receptor sequencing (scBCR‐seq), respectively (Table S2, Supporting Information). A scBCR‐seq library was not successfully constructed because of the limited number of B cells in some of the patients. After excluding CAR‐T cells, 166 140 high‐quality single cells were obtained for further analysis (Figure S1B–D and Table S2, Supporting Information).

All 37 single‐cell gene expression datasets were integrated, followed by nonlinear dimensionality reduction using Uniform Manifold Approximation and Projection (UMAP). Thirty clusters were identified based on canonical markers, representing diverse cell types, with a slightly higher enrichment of B cells and proliferating cells in the BM than in the PB, and *MME*‐positive B cell clusters 22 and 25 were not detected in the PB (Figure 1C, Figure S1E–G and Tables S3 and S4, Supporting Information). Compared with HDs, the mean proportions showed a reduction in B cells post‐5CAR and a decrease in B/NK/monocytes/dendritic cells (DCs) post‐7CAR. No statistical tests were performed because of the small sample size (Figure 1D, Figure S2A, Supporting Information).

The Augur score revealed that the T‐cell clusters in the 5CAR group exhibited pronounced gene expression changes compared with those in the HDs, T‐ALL, 7CAR, or 19+22CAR groups. Some B‐cell clusters (< 20; C25/22/18) contained too few cells for evaluation (Figure 1E, Figure S2B, Supporting Information). T‐ALL disease status and CAR therapy also affected gene expression in NK cells, monocytes, and DCs. T‐B, B‐B, and NK‐B cell interactions were reduced in the 5CAR group compared with those in the HDs, T‐ALL, 7CAR, and 19+22CAR groups (Figure S2C, Supporting Information).

### 2.2 Excessive T‐Cell Activation and Exhaustion in Patients after 5CAR Therapy

**Figure 2:**
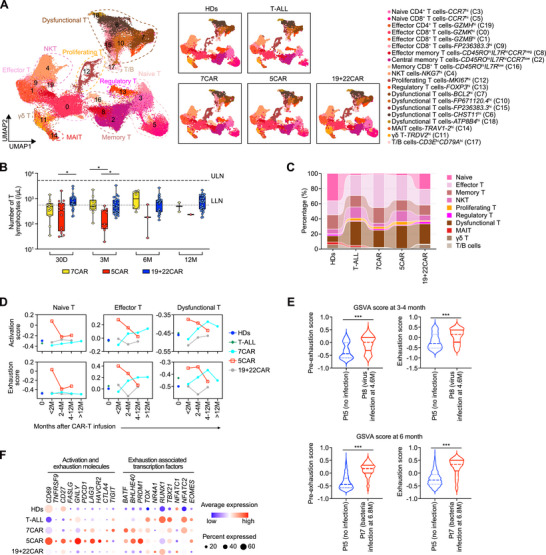
Excessive T‐cell activation and exhaustion in patients after 5CAR therapy. A) T‐cell sub‐clustering UMAP in all groups (left) and in each group (right). B) T‐cell counts in the peripheral blood of patients after 5CAR (NCT05032599), 7CAR (ChiCTR2000034762), and 19+22CAR therapies (NCT04340154). Dots represent individual patients. n = 13 in 7CAR, n = 14 in 5CAR, n = 27 in 19+22CAR at day 30 (30D); n = 10 in 7CAR, n = 9 in 5CAR, n = 27 in 19+22CAR at month 3 (3M); n = 6 in 7CAR, n = 3 in 5CAR, n = 30 in 19+22CAR at month 6 (6M); n = 2 in 7CAR, n = 1 in 5CAR, n = 29 in 19+22CAR at month 12 (12M). Two‐sided unpaired Kruskal‐Wallis test and subsequent Dunn's multiple comparisons test. C) Relative percentage of T‐cell clusters from all samples in each group. D) GSVA score of T‐cell activation and exhaustion at different time points in each T‐cell cluster, in each group. E) GSVA enrichment score of indicated features in samples from Pt5 and Pt8 patients 3–4 months after 5CAR treatment, and samples from Pt5 and Pt7 patients 6 months after 5CAR treatment. Two‐sided unpaired Mann‐Whitney test. F) Representative T‐cell activation and exhaustion molecule and exhaustion‐associated transcription factor gene expression in each group. *p < 0.05, ***p < 0.001. GSVA, Gene Set Variation Analysis; LLN, lower limit of normal; M, month; ULN, upper limit of normal; UMAP, Uniform Manifold Approximation and Projection.

T cells were reduced in number after 5CAR therapy and sub‐clustered into 20 clusters (**Figure** **2**A,B, Table S5, Supporting Information), including canonical naïve and effector subpopulations. Clusters 7/10/15/6/18 were classified as dysfunctional T cells based on minimal or absent effector molecules (e.g., granzymes) and no enrichment for exhaustion markers (e.g., *CTLA4*, *HAVCR2*, *TIGIT*, *LAG3*, and *PDCD1*) (Figure S3A,B, Supporting Information).

The percentage of naïve T cells was lower, whereas the percentages of proliferating and effector T cells were higher in the 7CAR and 5CAR groups than in the HDs (Figure 2C, Figure S3C,D, Supporting Information). The percentages of proliferating and dysfunctional T cells were higher, and the percentages of naïve T cells were lower in patients with T‐ALL than in those with HDs.

5CAR treatment significantly reduced *CD5* ^pos^ T cells, whereas 7CAR treatment reduced *CD7* ^pos^ T cells (Figure S4A, Supporting Information). Gene Ontology (GO) enrichment analysis revealed that T cells were activated post‐5CAR or post‐7CAR (Table S6, Supporting Information). Gene Set Variation Analysis (GSVA) showed that T cells post‐5CAR had higher levels of TCA cycle activity, activation, differentiation and exhaustion than the other groups, but lower naïve phenotype and most antiviral responses than HDs (Figure S4B, Supporting Information). The 7CAR group displayed less pronounced changes. Early after 5CAR treatment (< 2 months), activation and exhaustion levels were high but declined over time (Figure S4C, Supporting Information). In the 5CAR group, four patients had undergone prior CD7‐CAR (7+5CAR) therapy, and three received only CD5‐CAR (only‐5CAR) therapy. Enhanced activation and exhaustion scores were observed in both the 7+5CAR and only‐5CAR groups (Figure S4D, Supporting Information). In contrast, the 7CAR group showed no early changes in activation and exhaustion; instead, these features gradually increased over time, with the peak exhaustion score still lower than that of the early stage 5CAR group (Figure S4C, Supporting Information). This pattern was consistent across T‐cell subsets and was accompanied by metabolic changes (Figure 2D, Figure S4E, Supporting Information).

Chronic viral infections promote T‐cell exhaustion.^[^ ^22^ ^]^ In this study, early stage 5CAR samples were collected before infection (Figure 1B and Table S1, Supporting Information). Comparatively, samples from patients who developed infections had higher pre‐exhaustion and exhaustion scores before infection onset than those from infection‐free patients in the 5CAR‐treated group (Figure 2E), suggesting that higher exhaustion increases the risk of developing infection. Excessive T‐cell activation can accompany or drive exhaustion.^[^ ^23^, ^24^ ^]^ After 5CAR treatment, T cells exhibited higher expression of the activation marker *CD69* and exhaustion markers *PDCD1*, *LAG3*, and *HAVCR2* than those in the other groups (Figure 2F). Additionally, the 5CAR group showed high levels of the exhaustion‐associated transcription factors *BHLHE40*, *PRDM1*, and *NR4A1* (Figure 2F).^[^ ^25^ ^]^

Effector molecule levels in T cells were significantly increased early after 5CAR treatment compared with those in HDs, but decreased over time (Figure S4F,G, Supporting Information). In contrast, the levels of effector molecule gradually increased over time in patients treated with 7CAR therapy.

### 2.3 CD5 Deficiency was Associated with T‐cell Activation and Exhaustion In Vitro

Almost all samples (10/12) in the 5CAR group were CD5 protein‐negative (Table S7, Supporting Information). Non‐CAR T cells after 5CAR infusion contained *CD5* ^KO^ cells (edited by CRISPR/Cas9 at exon3) and natural CD5 protein‐negative cells.^[^ ^7^ ^]^ A critical question is whether *CD5* ^KO^ T cells function similarly to natural CD5 protein‐negative T cells. Therefore, we selected CD5 protein‐negative samples to investigate the characteristics of *CD5* ^KO^ (*CD5* mRNA^+^; exon3‐mutation) and *CD5* ^unedited^ (*CD5* mRNA^+^; exon3‐wildtype) cells in the scRNA data. T cells in the early stages after 5CAR treatment, regardless of *CD5* gene editing, showed higher activation and exhaustion than those post‐7CAR treatment (Figure S4H, Supporting Information).

Next, we investigated whether *CD5* deletion or the absence of natural CD5 protein directly drives T‐cell activation and exhaustion. In vitro, natural CD5^pos^, natural CD5^neg^, and engineered CD5^KO^ T cells were isolated or generated from HDs (Figure S5A, Supporting Information). CD5^KO^ T cells showed significantly higher CD69 and programmed cell death protein 1 (PD‐1) expression than CD5^pos^ T cells with or without Epstein‐Barr virus (EBV) peptide stimulation (Figure S5B,C, Supporting Information). CD69 expression was higher in CD5^neg^ cells than in CD5^KO^ or CD5^pos^ T cells following EBV peptide stimulation, whereas PD‐1 expression did not. LAG3 expression showed no significant differences among the three T‐cell types before and after stimulation (Figure S5D, Supporting Information). The exhaustion‐associated transcription factor B lymphocyte‐induced maturation protein 1 (Blimp‐1, encoded by the *PRDM1* gene) was upregulated in CD5^KO^ compared with that in CD5^pos^ T cells, whereas NR4A1 showed no change (Figure S5E,F, Supporting Information). Furthermore, Blimp‐1 knockdown in CD5^KO^ T cells resulted in PD‐1 downregulation (Figure S5G,H, Supporting Information). In bulk RNA‐seq, both CD5^KO^ and natural CD5^neg^ T cells exhibited slightly enhanced activation and exhaustion relative to CD5^pos^ T cells, with lower normalized enrichment scores in natural CD5^neg^ T cells than in CD5^KO^ T cells (Figure S5I, Supporting Information), suggesting that CD5^KO^ T cells had higher activation and exhaustion levels. Furthermore, *LAG3*, *CTLA4*, and *PDCD1* expression levels were higher in CD5^KO^ T cells than those in CD5^pos^ and CD5^neg^ T cells (Figure S5J, Supporting Information). Meanwhile, in vitro culture showed that CD5^KO^ T‐cell proliferation was lower than that of CD5^pos^ T cells (Figure S5K, Supporting Information). These data demonstrate that CD5 knockdown leads to T‐cell over‐activation and exhaustion, with CD5^neg^ T cells also contributing to these changes post‐5CAR. PD‐1 and LAG3 expression was higher in CD7^neg^ T cells than in CD7^pos^ T cells; however, the difference was not statistically significant under EBV peptide stimulation (Figure S5L, Supporting Information).

Upon PMA + ionomycin stimulation, IFN‐**γ** protein expression in CD5^KO^ T cells was comparable to that in CD5^neg^ and CD5^pos^ T cells (Figure S6A, Supporting Information). However, under EBV peptide stimulation, CD5^KO^ T cells produced less IFN‐**γ** protein than CD5^pos^ T cells (Figure S6B, Supporting Information), whereas *IFNG* mRNA levels did not decrease (Figure S6C, Supporting Information). CD5^KO^ and CD5^pos^ T cells produced similar levels of IFN‐**γ** when stimulated with cytomegalovirus (CMV) peptide (Figure S6D, Supporting Information). IFN‐**γ** secretion was slightly lower in CD7^neg^ than in CD7^pos^ T cells under stimulation, with no statistical difference (Figure S6E, Supporting Information). These results suggest that CD5 deficiency impairs the EBV‐associated T‐cell responses in vitro.

### 2.4 Decreased TCR Diversity and Pathogen‐Associated TCRs after 5‐ or 7CAR Therapy

**Figure 3:**
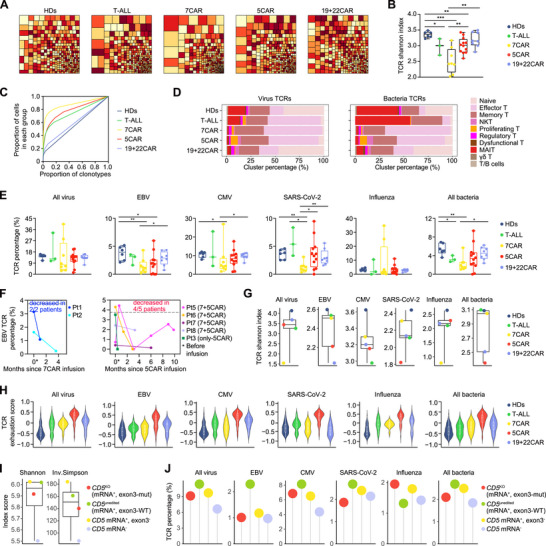
Decreased TCR diversity and pathogen‐associated TCRs after 5‐ or 7CAR therapy. A) Treemap plot of the top 400 TCR β‐chains, based on scTCR‐seq data. Each rectangle in a treemap plot represents a unique combination of VH‐D‐JH‐C genes, with size indicating relative frequency. B) TCR Shannon index of all T cells. Boxplots show median ± interquartile range (IQR), whiskers indicate full range, n = 6 in HDs, n = 3 in T‐ALL, n = 8 in 7CAR, n = 12 in 5CAR, n = 8 in 19+22CAR group. Two‐sided unpaired Mann‐Whitney test. C) Lorenz curve of clonotype proportion within a group versus total cell proportion within the same group. D) Each T‐cell cluster proportion in pathogen‐associated TCRs. E) Percentage of indicated pathogen‐associated TCRs from all patients and healthy donors. Boxplots show median ± IQR. The whiskers indicate the full range (top), or the 25th and 75th percentiles (bottom). n = 6 in HDs, n = 3 in T‐ALL, n = 8 in 7CAR, n = 12 in 5CAR, n = 8 in 19+22CAR group. Two‐sided unpaired Mann‐Whitney test. F) EBV‐associated TCR percentage before and after 7CAR or 5CAR treatment in the same individual patient. Different colors indicate different patients. G) TCR diversity of indicated pathogen‐associated TCRs from samples within 2 months. Boxplots show median ± IQR, whiskers indicate full range. H) GSVA score of T‐cell exhaustion of indicated specific T cells from samples within 2 months. White dots and vertical lines indicate median ± IQR. I) TCR diversity across different *CD5* gene editing states in T cells from patients receiving 5CAR therapy. *CD5* mRNA^+^ and exon3^−^ indicates *CD5* mRNA^+^ and exon3‐undetected T cells, where it was not clear whether exon3 was edited by gRNA. Boxplots show median ± IQR, whiskers indicate full range. J) Indicated pathogen‐associated TCR percentages across different *CD5* gene editing states in T cells from patients receiving 5CAR therapy. A‐J were determined by scTCR‐seq. *p < 0.05, **p < 0.01, ***p < 0.001. CMV, cytomegalovirus; EBV, Epstein‐Barr virus.

Based on the scTCR repertoire, 72 982 single cells representing 40 777 distinct clonotypes with paired TCR α‐ and β‐chains were identified (Table S8, Supporting Information). The 7CAR, 5CAR, and T‐ALL groups had lower TCR diversity than the HDs, whereas the 19+22CAR group maintained normal TCR levels (**Figure** **3**A,B). The 7CAR and 5CAR groups exhibited greater T‐cell clonal expansion than the HDs (Figure 3C). Reduced TCR diversity likely reflects either cell expansion or, more likely, a dramatic reduction in the T‐cell population.

Severe viral and bacterial infections were common during 5CAR or 7CAR therapy (Figure 1B).^[^ ^7^, ^8^, ^18^, ^19^, ^26^ ^]^ The unexpanded (n = 1) and expanded clones (n ≥ 2) maintained similar putative virus‐ or bacteria‐associated TCR percentages (Figure S7A, Supporting Information). All database‐annotated virus‐ and bacteria‐associated TCRs were primarily enriched in effector, naïve, memory T, and MAIT cells (Figure S7B, Supporting Information). In patients with 7CAR or 5CAR therapy, effector and memory T cells were the primary sources of these TCRs (Figure 3D). Compared with the HDs group, the 7CAR and T‐ALL groups had fewer bacteria‐associated TCRs (Figure 3E). The percentage of EBV/CMV/SARS‐CoV‐2‐associated TCRs was lower in the 7CAR group than in the HDs. The 5CAR group showed a reduced proportion of EBV‐associated TCRs. In addition, individual patient analyses revealed a progressive reduction in EBV‐associated TCRs after 5CAR treatment, which was consistent between the 7+5CAR and only‐5CAR groups (Figure 3F, Figure S8A, Supporting Information).

The diversity of each given pathogen‐associated TCR was then analyzed. Compared with the T‐ALL and HDs groups, the 7CAR group had a lower diversity of EBV/CMV/influenza/bacteria‐associated TCRs, whereas the 5CAR group had lower EBV/bacteria‐associated TCR diversity (Figure S8B, top, Supporting Information). In the early stage samples, the 5CAR group had a significantly lower diversity of EBV/CMV/SARS‐CoV‐2‐ or bacteria‐associated TCRs than the 7CAR group (Figure 3G). A similar change was observed when the 7+5CAR and only‐5CAR groups were analyzed separately (Figure S8C, Supporting Information). The exhaustion of T cells with these associated TCRs was higher in the 5CAR group than in the 7CAR or other groups (Figure S8B, bottom, Supporting Information), particularly in the early stages (Figure 3H).

*CD5* ^KO^ T cells from 5CAR‐treated patients had lower TCR diversity than *CD5* ^unedited^ T cells (Figure 3I). The proportion of EBV‐associated TCR in *CD5* ^KO^ cells was reduced by nearly half compared with that in *CD5* ^unedited^ T cells (Figure 3J).

Using in vitro isolated or generated natural CD5^pos^, CD5^neg^, and CD5^KO^ cells from HDs, bulk TCR‐seq revealed that CD5^neg^ T cells had lower percentages and diversity of various pathogen‐associated TCRs, including EBV‐associated TCR, than CD5^pos^ T cells in the absence of stimulation (Figure S9A,B, Supporting Information). After 2–7 days of EBV peptide stimulation, CD5^neg^ T cells showed reduced EBV‐associated TCR percentage and diversity (Figure S9C, Supporting Information), suggesting an intrinsic defect in the EBV response of these cells. Although CD5^KO^ T cells initially had a similar EBV‐associated TCR percentage as CD5^pos^ T cells, this declined after 7 days (Figure S9C, Supporting Information, top), likely due to the exhaustion of CD5^KO^ T cells (Figure S5J, Supporting Information). The TCR diversity in CD5^pos^ T cells was slightly higher than that in CD5^KO^ cells at 2 days, but decreased later to resemble CD5^KO^ cells, likely due to the preferential expansion of dominant clones (Figure S9C, bottom, and S9D, Supporting Information). Tetramer staining of three independent donors further showed a reduction in EBV‐specific tetramer^+^CD8^+^ T cells among CD5^KO^ T cells after 7 days of culture (Figure S9E, Supporting Information). These findings indicate that both CD5^KO^ and CD5^neg^ subpopulations contribute to the decline in EBV‐associated TCR percentages and diversity. Moreover, CD7^neg^ T cells displayed lower percentages and diversity of virus/bacteria‐associated TCRs than CD7^pos^ T cells (Figure S9F,G, Supporting Information), consistent with the reduced pathogen‐associated TCR frequencies and diversity in patients after 7CAR therapy.

### 2.5 Decreased B‐Cell Activation, Diversity Post‐5CAR, and Decreased Diversity Post‐7CAR

**Figure 4:**
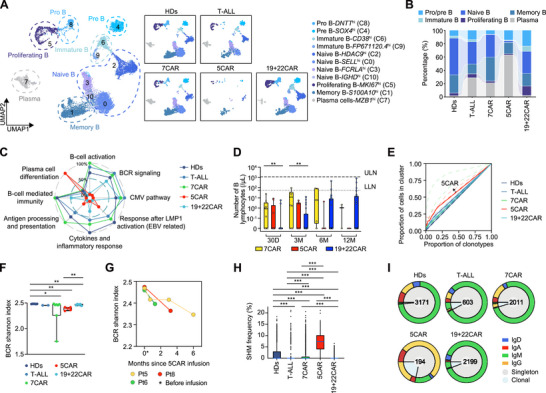
Decreased B‐cell activation, diversity post‐5CAR, and decreased diversity post‐7CAR. A) B‐cell sub‐clustering UMAP in all groups (left), and in each group (right). B) Relative percentage of B‐cell clusters from all samples. C) GSVA score of B‐cell characteristic pathways, normalized by all scored cells. D) B‐cell counts in the peripheral blood of patients after 5CAR (NCT05032599), 7CAR (ChiCTR2000034762), and 19+22CAR therapies (NCT04340154). Dots represent individual patients. n = 13 in 7CAR, n = 14 in 5CAR, n = 27 in 19+22CAR at day 30 (30D); n = 10 in 7CAR, n = 9 in 5CAR, n = 27 in 19+22CAR at month 3 (3M); n = 5 in 7CAR, n = 3 in 5CAR, n = 30 in 19+22CAR at month 6 (6M); n = 2 in 7CAR, n = 1 in 5CAR, n = 29 in 19+22CAR at month 12 (12M). Two‐sided unpaired Kruskal‐Wallis test and subsequent Dunn's multiple comparisons test. E) Lorenz curve of clonotype proportion within a group versus total cell proportion within the same group. No BCR was detected in the only‐5CAR group. The median value for each group, shown as a solid line, is used to represent the trend for that group, while the original data are shown as semi‐transparent dotted lines. F) BCR diversity. Boxplots show median ± IQR, whiskers indicate full range, n = 6 in HDs, n = 2 in T‐ALL, n = 7 in 7CAR, n = 5 in 5CAR, n = 5 in 19+22CAR group. Two‐sided unpaired Mann‐Whitney test. G) BCR Shannon before and after 5CAR treatment in the same individual patient. H) Frequency of somatic hypermutation based on scBCR‐seq. Boxplots show median ± IQR, whiskers indicate full range with excluding outliers. Two‐sided unpaired Mann‐Whitney test. I) ScBCR repertoires. Numbers indicate number of sequences. Singleton abundance was defined as 1; clonal abundance was > 1. *p < 0.05, **p < 0.01, ***p < 0.001. LLN, lower limit of normal; SHM, somatic hypermutation; ULN, upper limit of normal.

B cells are key components of the humoral immune system. We also examined the changes in B cells after CAR‐T cell treatment. Consistent with CD5 expression in B cells, 5CAR treatment rapidly reduced the number of *CD5* ^pos^ B cells in most patients (Figure S10A, Supporting Information). Sub‐clustering identified 11 B cell clusters (**Figures** **4**A, S10B and Table S9, Supporting Information). 5CAR patients showed a reduced relative proportion of naïve B cells and an increased proportion of plasma cells in B cells, both in the total and in BM samples (Figure 4B, Figures S10C,D, Supporting Information). These changes began early and may have been caused by high *CD5* expression in naïve B cells (Figures S10E and S11A, Supporting Information). Post‐7CAR, the relative percentage of plasma cells in B cells increased moderately, although to a lesser extent than that after 5CAR treatment. The subset composition remained unchanged after 19+22CAR treatment compared with that in HDs (Figure 4B, Figure S10C, Supporting Information).

GSVA revealed impaired activation and function (including CMV/EBV‐related response, antigen processing, and presentation), BCR signaling, and B cell‐mediated immunity in the 5CAR group compared with the 7CAR, T‐ALL, and HDs groups (Figure 4C, Figure S11B,C, Supporting Information). Notably, the plasma cell differentiation score was significantly higher in the 5CAR group. These changes were observed early after 5CAR treatment, and separate analyses of the 7+5CAR and only‐5CAR groups showed similar results (Figure S11D, Supporting Information). The 19+22CAR group showed some B cell impairment but no increase in the relative proportion of plasma cells.

In vitro, CD5^neg^ B cells expressed lower levels of the costimulatory molecule CD40 and the activation marker CD69 than CD5^pos^ B cells, which was consistent with the reduced mRNA levels (Figure S11E–G, Supporting Information). Furthermore, CD5^neg^ B cells secreted significantly less EBV IgG than CD5^pos^ B cells (Figure S11H, Supporting Information). These findings suggest that 5CAR therapy adversely affects both the quantity and functionality of B cells (Figure 4D).

Based on the scBCR repertoire, 8032 paired heavy‐ and light‐chain VDJ region sequences from 8806 single B cells met the filtering criteria (Table S10, Supporting Information). BCR diversity reduction and B‐cell clonal expansion were the most dramatic in the 5CAR group and worsened over time (Figure 4E–G, Figure S11I, Supporting Information). BCR diversity was also reduced in the 7CAR and 19+22CAR groups compared with that in the HDs. The frequency and count of somatic hypermutation^[^ ^27^ ^]^ were the highest in the 5CAR group (Figure 4H, Figure S11J, Supporting Information). The 5CAR group predominantly contained IgG‐secreting B cells, whereas the other groups predominantly contained IgM‐secreting B cells (Figure 4I).

Pseudotime analysis indicated that B cells in the 5CAR group predominantly followed fate 1, marked by plasma cell enrichment and exhaustion gene expression (Figure S12, Supporting Information).

Cell‐cell interaction analysis revealed that T‐B and B‐B cell interactions were markedly reduced in the 5CAR group, whereas they were maintained or increased in the T‐ALL and 7CAR groups (Figure S13A, Supporting Information). The key pairs diminished post‐5CAR included LGALS9‐ and MIF‐related interactions, which are important for B cell signaling and immune defense (Figure S13B, Supporting Information).^[^ ^28^, ^29^, ^30^, ^31^ ^]^

### 2.6 Perturbation of NK Cells and Myeloid Compartment

**Figure 5:**
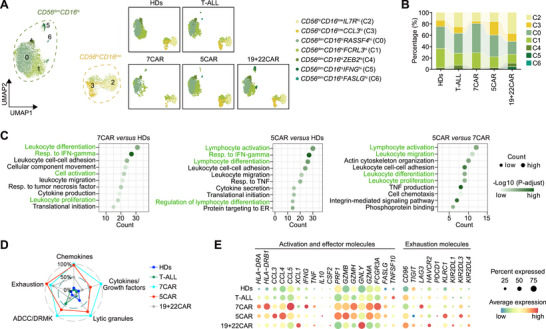
Reduced NK cell abundance post‐7CAR and enhanced activation post‐5CAR or post‐7CAR. A) NK cell sub‐clustering UMAP in all groups (left) and in each group (right). B) Relative percentage of NK cell clusters from all samples in each group. C) GO analysis of NK cells comparing 7CAR group with HDs, 5CAR group with HDs, and 5CAR group with 7CAR group. D) Radar plot showing GSVA score of NK cell characteristic pathways normalized by all scored cells from all samples. E) Expression of representative activation, effector, and exhaustion genes in each group. ADCC, antibody‐dependent cell‐mediated cytotoxicity; DRMK, death‐receptor‐mediated killing; Resp., response.

7CAR therapy reduced the number of CD7^pos^ NK cells (Figure S14A, Supporting Information),^[^ ^8^, ^32^ ^]^ however, the function of the remaining CD7^neg^ NK cells remains unclear. NK cells clustered into *CD56* ^hi^ *CD16* ^low^ (C2/3) and *CD56* ^dim^ *CD16* ^hi^ (C0/1/4/5/6) cell types (**Figures** **5**A, S14B and Table S11, Supporting Information). Compared with healthy donors, 7CAR had little effect on NK‐cell cluster proportions, whereas slight changes after 5CAR or 19+22CAR therapy may reflect bystander responses or lymphodepletion (Figure 5B, Figure S14C,D, Supporting Information). Compared with HDs, genes associated with NK cell activation/differentiation and response to IFN‐**γ** were increased in the 7CAR and 5CAR groups (Figure 5C). NK cells post‐5CAR showed higher levels of activation, migration, differentiation, and proliferation signatures than those in the 7CAR group (Figure 5C). NK cell cytotoxicity was significantly enhanced in the 7CAR and 5CAR groups compared with that in the HDs group (Figure 5D,E), and exhaustion was increased.

**Figure 6:**
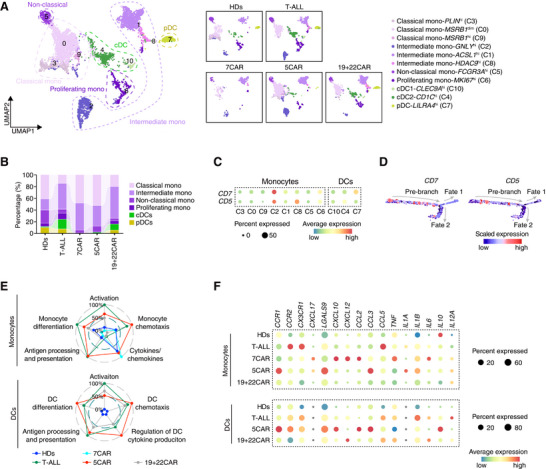
Complete disappearance of DCs post‐7CAR. A) Monocyte and dendritic cell sub‐clustering UMAP in all groups (left), and in each group (right). B) Relative percentages of monocyte and DC clusters from all samples in each group. C) Expression of *CD7* and *CD5* in each group. D) Expression of *CD7* and *CD5* in pseudotime trajectory. Dot size and color represent the average expression level. E) Radar plot showing GSVA score of monocyte or DC characteristic pathways normalized by all scored cells from all samples. F) Expression of representative chemokine and cytokine genes in monocytes or DCs in each group.

All monocytes and DCs were subdivided into 11 clusters: classical monocytes (C3/0/9), intermediate monocytes (C2/1/8), non‐classical monocytes (C5), proliferating monocytes (C6), conventional DCs (cDC1/cDC2; C10/4), and plasmacytoid DCs (pDCs; C7) (**Figure** **6**A, and S14E–G and Table S12, Supporting Information). Notably, the DCs disappeared in the 7CAR group (Figure 6A,B).

We investigated the reasons for DC disappearance. *CD7* and *CD5* mRNA were detected in DCs (considered mature DCs based on high levels of mature markers, such as *HLA‐DR*) in the T‐ALL and 19+22CAR groups, and *CD7* was also found in the DCs of HDs (Figure 6C, Figure S14H,I, Supporting Information). *CD7* and *CD5* were expressed early in the mono/DC differentiation pathway, which may explain the reduced pre‐branch percentage after 7CAR or 5CAR therapy (Figure 6D, Figure S15A,B, Supporting Information). Supporting the finding that BM CD34^+^CD7^+^ cells contain myeloid progenitors,^[^ ^33^ ^]^ we confirmed that some CD7^+^ or CD5^+^ DCs expressed CD34 (Figure S15C, Supporting Information). These data suggest that 7CAR or 5CAR therapy may eliminate mature DCs and disrupt mono/DC differentiation.

Monocytes and DCs showed increased activation, chemotaxis, antigen processing/presentation, and differentiation genes in the 5CAR and T‐ALL groups compared with the HDs (Figure 6E,F, Figure S15D, Supporting Information). Cytokine production (*IL1B* and *IL10*) increased in the 5CAR group of DCs (Figure 6F). In contrast, the 7CAR group displayed decreased monocyte activation and differentiation compared with the HDs and T‐ALL groups (Figure 6E).

## 3 Discussion

At the single‐cell level, this study revealed that both 7CAR and 5CAR therapies broadly disrupted the homeostasis and function of T, B, NK, and myeloid cells. Notably, the nature and extent of immune disruption differ substantially between the two therapeutic approaches. These findings provide evidence for understanding the risk of infection and suggest directions for further research on interventional approaches.

This study highlights the increased T‐cell exhaustion following 5CAR therapy. The T cells after 5CAR therapy were mostly composed of *CD5* ^KO^ and *CD5* ^neg^ cells. CD5, expressed on T cells, thymocytes, and B cells,^[^ ^34^ ^]^ has been shown to negatively regulate TCR signaling and thymocyte development, while promoting T‐cell differentiation and survival.^[^ ^27^, ^35^, ^36^, ^37^ ^]^ A previous study showed that CD4^+^ CD5^low^ T cells produce more IFN‐γ than CD4^+^ CD5^high^ T cells.^[^ ^38^ ^]^ In the present study, CD5^KO^ T cells produced less IFN‐γ protein than CD5^pos^ cells upon EBV peptide stimulation in vitro. However, no change in *IFNG* mRNA levels was found, suggesting post‐transcriptional regulation. Blimp‐1, a transcription factor that promotes T‐cell exhaustion during chronic infections,^[^ ^39^ ^]^ was upregulated in T cells post 5CAR therapy, and might play a role in promoting T‐cell exhaustion. These findings suggested that the functional impairment of T cells was at least partially attributable to the presence of CD5^KO^ T cells, which accounted for a significant proportion of peripheral nucleated cells in patients post 5CAR therapy.

The ability of TCRs and immunoglobulins to recognize antigenic peptides is a hallmark of the adaptive immune system. Single‐cell sequencing revealed a reduction in TCR diversity and EBV‐associated TCR following 5CAR therapy. Correspondingly, the level of EBV‐specific tetramer^+^ CD8^+^ T cells was lower in CD5^KO^ T cells than in CD5^pos^ T cells. A similar trend of reduced tetramer^+^ CD8^+^ T cells was observed in natural CD5^neg^ cells, although this difference did not reach statistical significance. The participation of CD5 in thymocyte development^[^ ^36^ ^]^ may contribute to the lower percentage of EBV‐associated TCR in CD5^neg^ T cells. Additionally, T‐cell exhaustion may exacerbate this reduction, with distinct patterns observed: CD5^neg^ T cells exhibited an inherently low percentage of EBV‐associated TCR, which was further diminished by exhaustion, whereas CD5^KO^ T cells displayed a progressive decline in EBV‐associated TCR, likely driven by exhaustion. Intriguingly, *CD5* knockout has been previously reported to enhance the antitumor efficacy and mitigate exhaustion of CAR‐T cells,^[^ ^40^ ^]^ suggesting disparate effects of CD5 on CAR and non‐CAR T cells. Furthermore, exhaustion signature was lower in patients without infection than in those with infection, further supporting the hypothesis that T‐cell exhaustion may increase infection risk by impairing pathogen‐associated T‐cell responses. This impaired T‐cell response to EBV aligns with clinical observations that two of 16 patients receiving 5CAR treatment died of EBV infection.^[^ ^7^ ^]^ However, TCR profiles associated with other pathogens, as identified by scTCR sequencing, require further validation through methods such as tetramer staining.

Single‐cell sequencing indicated that, unlike 5CAR therapy, 7CAR therapy caused less exhaustion of the T cells. However, 7CAR treatment impaired TCR diversity, consistent with previous findings from bulk TCR sequencing.^[^ ^16^ ^]^ This treatment also broadly reduced the proportions of TCRs associated with EBV, CMV, SARS‐CoV‐2, and bacteria. This reduction may partially contribute to the increased incidence of fatal infections observed following 7CAR therapy.^[^ ^16^ ^]^ The reduction of pathogen‐associated TCR may be attributed to the intrinsic properties of natural CD7^neg^ cells, as CD7^neg^ cells from HDs also exhibited lower proportions of pathogen‐associated TCRs than CD7^pos^ cells. Nevertheless, no significant reduction in IFN‐γ secretion was observed in peripheral blood mononuclear cells stimulated with EBV or CMV peptides following 7CAR treatment.^[^ ^16^ ^]^ These findings suggest that 7CAR may induce more diverse impairments in pathogen‐associated immune responses compared with 5CAR. Further research is required to elucidate the relationship between the downregulation of multiple pathogen‐associated TCRs and the increased incidence of infection.

This study investigated patients with T‐ALL treated with 5CAR or 7CAR therapies, using 19+22CAR therapy as a reference group. Our scRNA‐seq analysis revealed that TCR diversity and pathogen‐associated TCR in the 19+22CAR group remained largely comparable to those of HDs, suggesting that the observed reduction in TCR diversity following 5CAR and 7CAR therapies may contribute to the higher rates of severe infections. Furthermore, 5CAR and 7CAR therapies exhibited distinct kinetics of immune perturbation, potentially influencing the timing of infection. Specifically, T cells displayed high levels of exhaustion at earlier time points following 5CAR therapy, whereas exhaustion was initially low but progressively increased at later time points after 7CAR therapy. These patterns corresponded to the timing of severe infections, with a median onset of ≈2 months post‐infusion in patients receiving 5CAR therapy from a previous stem cell transplant donor, compared to ≈8 months in patients treated with 7CAR therapy.^[^ ^7^, ^16^ ^]^ These differences suggest that the risk of infection is associated with distinct immune perturbations induced by different T‐cell‐directed CAR therapies.

B‐cell impairments were more extensive following 5CAR therapy than 7CAR therapy, consistent with the expression of CD5, but not CD7, on B cells. BCR diversity was reduced after both 5CAR and 7CAR therapies, with a more pronounced reduction observed following 5CAR therapy. Additionally, compared with HDs, 5CAR therapy impaired B‐cell function and disrupted T‐B and B‐B interactions. In contrast, these were either maintained or enhanced following 7CAR therapy. As a critical component of the adaptive immune response, activated B cells differentiate into antibody‐secreting plasma cells and memory cells.^[^ ^41^ ^]^ Natural CD5^pos^ and CD5^neg^ B cells differ in their capacity to produce immunoglobulins that bind self‐antigens.^[^ ^42^ ^]^ Our analysis revealed that CD5^neg^ B cells exhibited lower expression of the co‐stimulatory molecule CD40^[^ ^43^ ^]^ than CD5^pos^ B cells. This reduced activation of CD5^neg^ B cells may contribute to decreased anti‐EBV IgG secretion observed in our in vitro analysis. B‐cell somatic hypermutation coincides with the formation of germinal centers and is essential for generating high‐affinity antibodies in plasma cells.^[^ ^44^, ^45^ ^]^ Following 5CAR treatment, the highest level of B cell SHM was observed. However, it remains unclear whether this increase in SHM results from the elimination of specific B‐cell subpopulations or the selective expansion of plasma cells. The functional impact of concurrently increased SHM and reduced BCR diversity on overall B‐cell immunity and clinical outcomes remains unknown, thus outlining a direction for future research.

This study also demonstrates changes in the composition and function of NK and myeloid compartments post 7CAR or 5CAR therapy. Consistent with the widespread expression of CD7 and rare expression of CD5 in NK cells,^[^ ^32^, ^46^ ^]^ 7CAR therapy reduced the percentage of NK cells among mononuclear cells, whereas 5CAR did not. However, no significant changes in NK cell subset composition were observed after 7CAR treatment. Although CD7 has been reported to enhance NK cell function,^[^ ^32^ ^]^ the cytotoxic pathway was upregulated in residual NK cells (mostly CD7^neg^) post 7CAR therapy. The role of CD7 in NK cell function requires further investigation.

DCs are essential for antigen presentation and the initiation of adaptive immunity.^[^ ^47^ ^]^ Consistent with our in vitro findings demonstrating CD7 and CD5 expression on distinct DC subpopulations, 7CAR therapy eliminated DCs, potentially due to targeting of the CD34^+^ progenitor population, which warranted further investigation. In contrast, 5CAR therapy caused a less pronounced reduction in DCs. CD1c^+^ CD5^+^ DCs in the skin and lymph nodes have been reported to promote robust T‐cell proliferation and virus‐specific responses.^[^ ^34^ ^]^ However, no significant functional impairment was observed in DCs post 5CAR therapy, which may be explained by a tissue‐specific role for CD5^+^ DCs.

Monocytes, critical components of innate immunity, differentiate into macrophages and dendritic cells. CD7 was found to be expressed on monocytes under nutritional stress, whereas the expression of CD5 on monocytes was not reported.^[^ ^48^ ^]^ Unlike a previous study that reported a reduced CD14^+^ monocyte subpopulation after 7CAR treatment,^[^ ^21^ ^]^ we observed a slight decrease in the percentage of proliferating monocytes with minimal CD14 expression. These discrepancies may reflect methodological or patient‐specific differences between studies. Compared with 19+22CAR, monocyte activation was elevated after 5CAR therapy but reduced after 7CAR therapy. Caution is warranted in interpreting these results, as monocyte numbers and function may also be modulated by cytokines and other non‐specific effects of CAR‐T cells. It could not be excluded that the alteration of monocytes is a secondary effect of changed function of non‐CAR T cells. Nevertheless, peak expansion of CD5‐ or CD7‐directed CAR‐T cells was not associated with the occurrence of CRS, ICANS, or severe infection in our previous studies.^[^ ^7^, ^8^, ^16^, ^49^ ^]^ The impact of CAR‐T cell expansion kinetics on immune dysfunction, including T‐cell alterations, monocyte activation, and infection risk, requires further investigation.

Lymphodepletion prior to CAR‐T cell therapy may also contribute to alterations in immune cell populations in patients. A previous study on CD19‐directed CAR‐T cell therapy showed that in CAR‐negative populations, the proportions of monocytes and DCs were higher after lymphodepletion chemotherapy than in healthy donors, whereas the proportions of CD8^+^ T cells, NK cells, and B cells were lower.^[^ ^50^ ^]^ Inclusion of samples from patients treated with 19+22CAR could provide insights into the target‐specific effects of 7CAR and 5CAR therapies.

This study has some limitations. First, in addition to lymphocyte aplasia, factors, such as disease progression and treatment may contribute to high infection rates.^[^ ^51^ ^]^ Second, due to limited sample availability, neither CAR‐T cells nor CAR‐naïve B‐ALL samples were subjected to sequencing analysis. Third, owing to the limited sample size, age‐stratified analyses were not performed, nor were there in vitro assays of antigen‐specific T‐cell activation upon TCR stimulation based on the patient samples. Fourth, donor cells may induce GVHD and inflammatory responses, which must be considered when interpreting these results. Finally, pathogen specificity was inferred from single‐chain (CDR3α or CDR3β) database matches rather than from the gold standard of paired αβ TCR data, and HLA restrictions could not be comprehensively evaluated because the public TCR databases used for annotation contained heterogeneous HLA information. Therefore, potential cross‐reactivity and limited database validation could affect the accuracy of our pathogen‐associated TCR assignments, and the results should be interpreted as indicative of repertoire‐level trends rather than definitive antigen‐HLA specificities. Given that VDJdb catalogs antigen‐specific TCRs recognizing defined epitope‐MHC complexes, while McPAS‐TCR compiles pathology‐associated sequences, the matched TCRs in this study are designated as pathogen‐associated.^[^ ^52^, ^53^ ^]^ Further studies using paired‐chain sequencing and functional assays are warranted. While we acknowledge the technical limitation of single‐chain TCR sequencing, this method has been used to yield biologically insightful findings in other contexts.^[^ ^54^, ^55^ ^]^ Coupled with our stringent matching criteria, which minimize background noise, we believe that our approach may still suggest shifts in clonal frequency and reflect meaningful trends.

In conclusion, our findings offer a single‐cell‐resolution molecular atlas of immune cell dysregulation following 5CAR and 7CAR therapies. This paves the way for deeper investigations into the mechanisms involved in preventing infection in CAR‐T therapies.

## 4 Experimental Section

### Study Design

This study investigated immune dysfunction in T‐ALL patients receiving 5CAR or 7CAR therapies. Patient samples were collected from treated individuals and compared with controls, including B‐ALL patients receiving 19+22CAR therapy, untreated T‐ALL patients, and healthy donors. Multicolor flow cytometry sorting was used to isolate mononuclear or lymphocyte subsets (T, B, and NK cells). scRNA‐seq analyzed immune cell composition, activation states, effector function, exhaustion levels, intercellular communication, and differentiation trajectories via pseudotime analysis. scTCR‐seq and scBCR‐seq were performed to assess changes in TCR/BCR diversity and shifts in pathogen‐associated TCR clonotypes post‐CAR therapy. In vitro assays, including flow cytometry and ELISA, evaluated immune activation, IFN‐γ secretion, exhaustion‐associated transcription factor expression, tetramer^+^ T cell proportions, and EBV‐IgG levels. These experiments were conducted on T and B cells lacking CD5 protein, either naturally negative or CRISPR/Cas9‐edited, with or without viral stimulation.

### Flow Cytometry

BM and PB mononuclear cells were isolated by Ficoll‐Paque centrifugation and stained with FITC anti‐human CD45 (304 006, clone HI30, BioLegend, USA), PE/Cyanine7 anti‐human CD5 (364 008, clone L17F12, BioLegend, USA), and PE anti‐human CD7 (343 106, clone CD7‐6B7, BioLegend, USA), APC/Cyanine7 anti‐human CD3 (300 318, clone HIT3a, BioLegend, USA), PE anti‐human CD19 (302 208, clone HIB19, BioLegend, USA), APC anti‐human CD19 (302 212, clone HIB19, BioLegend, USA), PerCP/Cyanine5.5 anti‐human CD56 (318 322, clone HCD56, BioLegend, USA). To deplete tumor cells (based on clinical data) and/or CAR‐T cells, CD19 and CD22 CAR‐T cells were labeled with recombinant human CD19 (RP01307, Pro20‐Lys291, ABclonal, China) and CD22 (RP00580, Asp20‐Arg687, ABclonal, China) proteins with His tag, respectively, followed by anti‐His (362 605, clone J095G46, BioLegend, USA), and CD5 and CD7 CAR‐T cells were labeled with recombinant human chimeric CD5‐Fc (CD5‐H5253, Arg25‐Pro372, ACROBiosystems, China) and CD7‐Fc (CD7‐H5253, Ala26‐Pro180, ACROBiosystems, China) proteins, respectively, followed by anti‐human IgG Fc (410 712, clone M1310G05, BioLegend, USA).

CD11c^+^ DCs were detected as live LIN (CD3, CD19, CD56)^−^ CD14^−^HLA‐DR^+^CD11c^+^ cells and pDCs were detected as live LIN (CD3, CD19, CD56)^−^ CD14^−^HLA‐DR^+^CD11c^−^CD123^+^ cells.

After staining with anti‐CD5 and anti‐CD3 antibodies, T cells were fixed and permeabilized using the BD IntraSure^TM^ Kit, and then stained with Alexa Fluor 647 anti‐Blimp‐1 (565 002, clone 6D3, BD Biosciences, USA) or anti‐Nur77/NR4A1 (3960T, clone D63C5, Cell Signalling Technology, USA) primary antibody, followed by anti‐rabbit IgG secondary antibody (406 403, clone Poly4064, BioLegend, USA). Flow cytometry data were recorded on FACSCanto II or FACSAria III (BD Biosciences, USA) and analyzed using FlowJo software (Tree Star, OR, USA).

### Genomic Disruption of CD5 in Primary T Cells

Guide RNA (gRNA) sequence for CD5 (CGGCTCAGCTGGTATGACCC AGG) was used. CD3^+^ T cells were isolated from HD PBMCs using the EasySep Human T Cell Enrichment Kit (19 051, STEMCELL, Canada) and cultured in T cell medium (10 981, STEMCELL, Canada) containing 10% fetal bovine serum (FBS, 16 000 044, Gibco, USA), 100 U mL^−1^ IL‐2 (GMP‐TL906‐0100, Beijing Tonglihaiyuan, China). T cells were activated with anti‐CD3/CD28/CD2 (10 970, STEMCELL, Canada) and cultured at 37 °C in a 5% CO_2_ incubator. After 48 h, 1 × 10^6^ activated T cells were resuspended in 20 µL Cas9/gRNA ribonucleoproteins (RNPs) consisting of 1 µL gRNA (0.1 nmol µL^−1^, GenScript, China) and 1 µL Cas9 protein (5 µg µL^−1^, A50576, Invitrogen, USA). Electroporation was performed according to the P3 Primary Cell 4D‐Nucleofector X Kit (Lonza, Switzerland) instructions. CD5^pos^ and CD5^neg^ T cells were electroporated with control gRNA (GTGTAGTTCGACCATTCGTG). Knockdown efficiency was determined by flow cytometry at 72 h after electroporation.

### Culture and Induction of Human Monocyte‐Derived Dendritic Cells

CD14^+^ monocytes were isolated from PBMCs of HDs using CD14 MicroBeads (130‐050‐201, Miltenyi, Germany) and cultured in medium containing 100 ng mL^−1^ hGM‐CSF (RP00094, ABclonal, China) and 20 ng mL^−1^ hIL‐4 (RP00995, ABclonal, China).^[^ ^56^ ^]^ Immature DCs were harvested on day 6 and analyzed by flow cytometry. Poly‐IC (25 µg mL^−1^, P0913‐10MG, Sigma, Germany) and EBV peptides or CMV peptides (100 peptides with 2 µg mL^−1^ per peptide, 3641‐1 or 3619‐1, Mabtech, Sweden) were then used to induce mature DCs.^[^ ^57^ ^]^ Flow cytometry was used to determine phenotype of mature DCs.

### Generation of EBV‐Specific CTLs

CD5^pos^, CD5^neg^, and CD5^KO^ T cells were cocultured with mature DCs in a 100:1 ratio under EBV peptide stimulation (100 peptides with 2 µg mL^−1^ per peptide, 3641‐1, Mabtech, Sweden). EBV peptides and EBV peptides‐pulsed DCs were added to cocultures every 48 h, and EBV‐specific CTLs were obtained on day 2 or 7.^[^ ^58^ ^]^ In each experiment batch, CD5^pos^, CD5^neg^, CD5^KO^ T cells, and mature DCs were generated from the same donor. The HLA alleles covered by the EBV peptide pool include: HLA‐A*01, 02, 03, 11, 24, 25, 26, 68, HLA‐B*07, 08, 15, 18, 23, 25, 26, 27, 35, 38, 40, 44, 55, 57, 58, HLA‐DRB1*01, 03, 04, 07, 08, 11, 13, 15, 16, HLA‐DRB3*01, 02, HLA‐DRB4*01, HLA‐DRB5*01, HLA‐DPB1*104, 04, HLA‐DPB4*01, and HLA‐DQB1*06, 07.

The activation and exhaustion phenotypes, as well as the cytokine secretion and transcription factor expression, in T cells stimulated with or without EBV peptides for 2 days, were measured by flow cytometry with the following antibodies: anti‐CD69 (310 926, clone FN50, BioLegend, USA), anti‐PD‐1 (329 904, clone EH12.2H7, BioLegend, USA), anti‐LAG‐3 (369 308, clone 11C3C65, BioLegend, USA), anti‐IFN‐γ (502 526, clone 4S.B3, BioLegend, USA), anti‐NR4A1 (3960T, clone D63C5, Cell Signalling Technology, USA), and anti‐Blimp‐1 (565 002, clone 6D3, BD Biosciences, USA).

After 7 days of EBV peptide stimulation, HLA‐A 24:02 EBV Mix Tetramer‐PE (TS‐M009‐1, MBL, Japan) was used to label EBV‐specific CTLs: after FcR blocking (422 301, BioLegend, USA), 10 µL Tetramer was used for an assay of 1 × 10^6^ cells/100 µL at 4 °C in the dark. HLA‐A genotypes were determined by high‐resolution HLA genotyping with Sequence Based Typing method, and IMGT/HLA database was considered in the typing. Samples with HLA‐A 24:02 genotype were used for Tetramer labeling.^[^ ^59^, ^60^ ^]^

### Intracellular Cytokine Staining

To detect IFN‐γ expression, T cells were stimulated with phorbol myristate acetate (PMA, 50 ng mL^−1^, CS0001, Multi Sciences, China) and ionomycin (500 ng mL^−1^, CS0002, Multi Sciences, China), or EBV peptides or CMV peptides (100 peptides with 2 µg mL^−1^ per peptide, 3641‐1 or 3619‐1, Mabtech, Sweden) with mature DCs, and then detected using Transcription Factor Staining Buffer Set after 4 h of GolgiStop (554 724, BD Biosciences, USA) addition.^[^ ^61^, ^62^ ^]^ The HLA alleles covered by the CMV peptide pool (3619‐1, Mabtech, Sweden) include: HLA‐A*01, 02, 03, 11, 23, 24, 26, 30, HLA‐B*07, 08, 18, 27, 35, 40, 41, 44, 57, 58, 60, HLA‐C*07, HLA‐DRB1*01, 03, 04, 05, 07, 08, 11, 15, 20, 24, 53, HLA‐DPw2, HLA‐DQB, and HLA‐DP3*14, 20.

### Enzyme‐Linked Immunosorbent Assay

PBMCs were separated on Ficoll‐Paque, and human CD5^pos^ and CD5^neg^ B cells were isolated from PBMCs by flow cytometric sorting. B cells were cultured in Iscove's modified Dulbecco's medium (IMDM, 12 440 053, Gibco, USA) with 10% FBS (16 000 044, Gibco, USA), in the presence of IL‐4 (2 ng mL^−1^, 78 045.1, STEMCELL, Canada), CD40 ligand (2 µg mL^−1^, 78 162, STEMCELL, Canada), and insulin‐transferrin‐selenium (41 400 045, Invitrogen, USA) for 2 weeks.^[^ ^63^ ^]^ A total of 100 peptides at a concentration of 2 µg mL^−1^ per peptide (3641‐1, Mabtech, Sweden) were added three times every 4 days. EBV‐specific IgG in culture supernatants was detected using an ELISA kit (FT‐P33200R, Shanghai Fantai, China) according to the manufacturer's instructions.

### Proliferation Assay

CD5^pos^ and CD5^KO^ T cells were cultured at 1 × 10^4^ per well in a 96‐well flat plate. The culture medium was changed by half every day. The number of T cells was counted with counting beads (424 902, BioLegend, USA) every 3 days for 30 days.

### Small Interfering RNA Transfection

Negative control siRNA (sequence: 5′‐UUCUCCGAACGUGUCACGUTT‐3') and siRNA targeting Blimp‐1 (sequence: 5′‐GCAACUGGAUGCGCUAUGUTT‐3′) were synthesized by GENCEFE Biotech (China). 40 pmol siRNA at a final concentration of 80 nM and polyethyleneimine mixture (1:3) were added to 2 × 10^5^ CD5^KO^ T cells in a 24‐well plate. The culture medium was changed after 6–8 h. PD‐1 and Blimp‐1 expression were detected after 2 days.

### Single‐Cell Sorting and Processing of Single Cell RNA‐seq, TCR‐seq, and BCR‐seq

On the basis of FACS analysis, tumor and CAR‐T cells were depleted, and DAPI^−^CD45^+^ cells or DAPI^−^CD45^+^CD3^+^ T cells, DAPI^−^CD45^+^CD19^+^ B cells, and DAPI^−^CD45^+^CD56^+^ NK cells were sorted. The sorted cells were resuspended in FACS buffer (0.04% BSA phosphate buffered saline) and processed directly for scRNA‐seq, scTCR‐seq, and scBCR‐seq library preparation, following the 10× Genomics manufacturer's protocol.

### scRNA‐seq Library Construction and Sequencing

In accordance with the manufacturer's guidelines, scRNA‐seq libraries were generated using the 10× Chromium Single Cell 5′ Platform (10× Genomics, USA). To generate single‐cell gel beads in emulsion, a single‐cell suspension was briefly loaded onto a chromium microfluidic chip. The single cell 5ʹ gene expression libraries were constructed using the Chromium Next GEM Single Cell 5′ Kit v2 (1 000 265, 10× Genomics, USA), while the single cell V(D)J enriched libraries were generated using the Chromium Single Cell Human TCR Amplification Kit (1 000 252, 10× Genomics, USA) or Chromium Single Cell Human BCR Amplification Kit (1 000 253, 10× Genomics, USA). Subsequently, the Agilent 2100 Bioanalyzer System was used to assess the quality of the libraries, and the Illumina NovaSeq 6000 Platform (Illumina, USA) was used for sequencing the data.

### Single‐Cell RNA‐Seq Analysis

To further identify and exclude CAR‐T cells within individual single‐cell transcriptome sequencing samples, the CAR‐T cell specific sequences (defined as specific single‐chain fragment variable and/or lentiviral vector sequences) were integrated into the human reference genome GRCh38‐2020‐A. These added sequences had no similar homologous sequences in the human genome. Subsequently, CellRanger v7.1.0 Software (10× Genomics, USA) was used to align each sample's sequencing data to the reconstructed human reference genome, facilitating the cell identification and expression matrix construction.

Quality control was performed on the expression matrices of each sample, using Seurat v4.3.0 software (RRID: SCR_01 6341). Quality control criteria were as follows: 1) cells expressing ≥ 200 genes and ≤ 8000 genes were retained, 2) cells with mitochondrial gene expression less than 20% were retained, and 3) genes detectable in at least 3 cells were retained. The DoubletFinder v2.0.2 package (RRID: SCR_01 8771) was used to eliminate doublets (≥ 2 cells in a single oil droplet).

The Canonical Correlation Analysis (CCA) algorithm, implemented in the Seurat software, was used to integrate multiple sample datasets. The integrated data assay was then scaled in Seurat using the ScaleData function. Principal Component Analysis (PCA) was conducted on the top 2000 highly variable genes to reduce the linear dimensionality. The first 50 PCs were used in the Seurat function FindNeighbors and FindClusters, and the resolution for identifying cell clusters was 0.6. The resulting cell clusters were visualized and investigated using UMAP. Cluster‐enriched genes were identified by the Seurat function FindAllMarkers, and the minimum percentage (min.pct) set was 0.25.

### Functional Enrichment Analysis

Gene Ontology (GO) functional enrichment analysis was used to investigate the functional significance of differentially expressed genes, using the R package clusterProfiler v3.14.0 (RRID: SCR_01 6884). This work utilized the following parameters to ensure comprehensive and robust identification of enriched GO terms: pvalueCutoff = 1, qvalueCutoff = 1, and pAdjustMethod = “BH.” Enriched GO terms with q‐values (adjusted p‐values) below 0.05 were considered to be significant.

### Quantification of CD45 Isoform Expression

CD45RA and CD45RO isoforms were used as markers to define T‐cell subclusters, and the method described previously was used to quantify CD45 isoform expression.^[^ ^64^ ^]^ The critical splicing events that distinguish between the CD45RA and CD45RO isoforms take place at the 5′ end of the gene (exons 4 to 7). To quantify the expression levels of CD45 isoforms, this work employed a model called CD45er. Following the official instructions (https://github.com/getzlab/10x‐cd45‐isoform‐quantification), this work analyzed the BAM files for each sample using CD45er. This tool calculates the probability for each read aligning to CD45, indicating its probability of belonging to one of the specific isoforms: RAX, RABX, RBX, RBCX, RABCX, or RX. The overall probability for CD45RA was determined by summing the probabilities of reads associated with the RAX, RABX, and RABCX isoforms. For CD45RO, the probability was indicated by the value in the RX column. Finally, this work aggregated these probabilities using cellular barcodes to generate the final counts employed in further subsequent analyses.

### Single Cell Pseudotime Analysis

Single‐cell pseudotime trajectory analysis was performed using Monocle 2 v2.26.0. The initial root state (t = 0) of the trajectory was determined by CytoTRACE v0.3.3 software (RRID: SCR_02 2828), which identifies the least differentiated cells based on transcriptional diversity. Genes exhibiting high dispersion across cells were selected as ordering features to minimize technical noise and prioritize biologically relevant genes with dynamic expression patterns. Cells were then ordered along a pseudotemporal continuum using reversed graph embedding. Dimensionality reduction and trajectory inference were implemented through the DDRTree algorithm, which simultaneously learned a reduced‐dimensional space (default dimensions = 2) and constructed a principal graph represented as a minimum spanning tree (MST) to model complex branching structures. Finally, branch‐dependent genes exhibiting significant expression changes across trajectory bifurcation points were identified using Branch Expression Analysis Modeling (BEAM; RRID: SCR_0 07258).

### Cell‐Cell Interaction Analysis

Intercellular communication networks were systematically inferred and analyzed using CellChat v2.1.0 software (RRID: SCR_02 1946), a computational framework leveraging curated databases of experimentally validated ligand‐receptor (L‐R) interactions. The analysis incorporated all major signaling categories within the CellChatDB database. For each identified cell type, CellChat first calculated communication probabilities by integrating the expression levels of ligands and receptors with established interaction stoichiometries, while accounting for co‐expression patterns within interacting cell pairs. Probabilistic values were derived through a permutation‐based null model (n = 1000 permutations) to estimate significance. To rigorously compare communication strengths between distinct cell types, the netVisual_bubble function was employed.

### Single‐Cell V(D)J Sequencing Analysis

To generate the single‐cell V(D)J sequences and annotate each sample library, the single‐cell V(D)J sequencing data of each sample were aligned to Human V(D)J reference (GRCh38) using CellRanger VDJ v4.0.0 software. The initial contig annotations provided by CellRanger VDJ, which contained high‐level annotations of each high‐confident cellular contig, were further filtered using the scRepertoire v1.8.0 (https://github.com/ncborcherding/scRepertoire) package to retain cells with high‐quality V(D)J information. The filtering criteria were as follows: 1) Cells must contain both heavy and light chains information for BCRs (or α and β chains information for TCRs), while cells with only one chain information were discarded. 2) In cases where a cell had multiple heavy or light chains for BCRs (or α and β chains for TCRs), this work retained the chain information with the highest UMI or read count. 3) Only cells with high‐quality V(D)J information that were also detected in the single‐cell transcriptome sequencing were retained. Clonotypes were identified based on the nucleotide sequence of the CDR3 region and the VDJC gene comprising, using the “strict” definition in scRepertoire nomenclature. For determining BCR clonotypes, a stringent definition utilizing the normalized Levenshtein edit distance of CDR3 nucleotide sequences and V‐gene usage was applied. This approach allows for the grouping of BCRs derived from the same progenitor that may have undergone mutations during somatic hypermutation and affinity maturation. The default similarity threshold for grouping was set at 0.85, calculated using the formula: threshold(s,t) = 1 – Levenshtein(s,t)/((length(s)+length(t)) / 2). Percent of unique clonotypes were obtained using the quantContig function. The clonotype expansions were categorized based on the relative frequency and defined as follows: Hyperexpanded (0.1 < X ≤ 1), Large (0.01 < X ≤ 0.1), Medium (0.001 < X ≤ 0.01), Small (1e‐04 < X ≤ 0.001), and Rare (0 < X ≤ 1e‐04), where X is the relative frequency of the clonotype. Integration with the Seurat object was executed using the combineExpression function. Shannon's entropy was calculated using the following formula: ‐∑(clonotype frequency * loge(clonotype frequency)) using the clonalDiversity function. All functions were executed with the exportTable = T setting to obtain a comprehensive table of results.

See Supporting Information for further details.

### Gene Set Variation Analysis

To further explore the functional characteristics of different cell populations, this work conducted GSVA on scRNA‐seq data, utilizing the GSVA v1.46.0 package in R. The gene set used was detailed in Table S13, Supporting Information.

### Bulk RNA Sequencing and Analysis

Total RNA from T cells was extracted with Trizol (Invitrogen). For bulk RNA‐seq, the quality of total RNA was assessed by the Agilent 2100 Bioanalyzer (Agilent Technologies, USA) and sequencing was performed using Illumina HiSeq X platform at Novogene (China). GSEA analysis was carried out using GSEA software (4.3.3; RRID: SCR_0 03199). The DESeq2 R package (1.20.0; RRID: SCR_01 5687) was used for differential expression analysis.

Bulk TCR‐seq sequencing and analysis: T cells were stimulated with or without EBV peptide. Then, RNA from these T cells was extracted and subjected to TCR sequencing in Beijing Gobroad Boren Hospital (China) and BIG Tech (China). TCR β chain CDR3 regions were amplified using multiplex Polymerase Chain Reaction with primers annealing to V and J segments and sequenced by the MGISEQ‐2000 platform, and also using the OncomineTM TCR Beta‐LR Assay and sequenced in Ion Torrent S5 platforms with single end 400 bp strategy. For the analysis of bulk TCR‐seq repertoire data, this work employed the MiXCR v4.3.2 software with the built‐in workflows oncomine‐human‐tcrb‐lr‐full‐length or ampliseq‐tcrb‐plus‐cdr3, using default parameters. These workflows were utilized for quality control, alignment, and assembly of the sequencing reads, identification of distinct clonotypes, and quantification of these clonotypes, while also extracting the CDR3 sequences of the beta chains for each clonotype. Subsequently, the R package immunarch v1.0.0 was used for the annotation of the CDR3 sequences of the beta chains for each clonotype. The TCR immune receptor database used for this annotation was consistent with the one employed for the single‐cell TCR‐seq (scTCR‐seq) analysis, ensuring a standardized and comparable approach across different datasets.

### Statistical Analysis

For the sequencing data, the statistical analyses and graphics production were performed using R (v.3.14.0) and RStudio (v.4.1.3). For experimental data, this work performed statistical analyses and generated graphs using GraphPad Prism (v.9.0, GraphPad Software, USA). No data outliers were excluded from these analyses. This work used two‐sided Kruskal‐Wallis (subsequent Dunn's multiple comparisons test) and Mann‐Whitney tests for unpaired three‐group and two‐group comparisons, respectively. A two‐sided one‐way repeated‐measures (RM) ANOVA (subsequent Tukey's multiple comparisons test) with Geisser‐Greenhouse correction and paired *t*‐test were used for paired three‐group and two‐group comparisons, respectively. Data are presented as the mean ± standard error of the mean^[^ ^11^ ^]^ or the mean ± standard deviation (SD), depending on the experiment. Unless otherwise specified, all experiments were performed at least three times. The number of donors (n) for each experiment is provided in the figure legends. Boxplots show the median and interquartile range (IQR), with whiskers indicating the full range. Statistical significance was defined as *p* < 0.05.

### Ethics Approval Statement

All patients were enrolled in clinical trials conducted at Beijing GoBroad Boren Hospital, Beijing, China (NCT04689659, ChiCTR2000034762, NCT05032599, NCT04340154, and Ethic ID: KY2024‐001‐001). All HDs were from the Institute of Hematology & Blood Diseases Hospital, Chinese Academy of Medical Sciences & Peking Union Medical College (IHCAMS). The trials adhered to the tenets of the Declaration of Helsinki, and the protocol was approved by the Institutional Review Board of Beijing GoBroad Boren Hospital and IHCAMS. All patients and HDs have provided written informed consent.

## Conflict of Interest

The authors declare no conflict of interest.

## Author Contributions

Y.L., H.Z., K.T., Y.W., and H.D. contributed equally to this work. Y.L. and X.F. conceived the project. Y.L. and X.F. designed the experiments. Y.L., H.Z., K.T., and L.S. performed the experiments; Y.L., H.Z., Y.W., W.Q., and H.D. analyzed the single‐cell sequencing data. All authors contributed to the interpretation of the results; J.P. provided clinical samples; Y.L., H.Z., K.T., Y.W., H.D., J.S., E.J., and J.P. and X.F. wrote the manuscript and provided comments for revision. Y.L., E.J., J.P., and X.F. supervised the project.

## Supporting information



Supporting Information

Supplemental Table

## Data Availability

The raw sequence data reported in this paper have been deposited in the Genome Sequence Archive in National Genomics Data Center,^[^ ^65^, ^66^ ^]^ China National Center for Bioinformation/Beijing Institute of Genomics, Chinese Academy of Sciences, with accession number HRA008086 (https://ngdc.cncb.ac.cn/gsa‐human). These data will be made available for scientific research upon request, in compliance with legal requirements to protect the privacy of human patients. All other data supporting the findings of this study are available from the corresponding author upon reasonable request. The original code for analysing sequence data has been posted to GitHub, and is available at https://github.com/zhx17812091205/sc‐seq‐for‐CD5‐CAR‐T‐therapy.
