# Supplementary material for: Single‐Cell Dissection Reveals Immune Dysregulation After CD5 or CD7‐Directed Chimeric Antigen Receptor T‐Cell Therapy
Source: Adv Sci (Weinh). 2025 Nov 25;13(8):e09259. doi: 10.1002/advs.202509259 (PMC12884813; doi:10.1002/advs.202509259)
Supplement: Supplementary file 1 — Supporting Information [file ADVS-13-e09259-s002.pdf]

# Supporting Information

**Single-Cell Dissection Reveals Immune Dysregulation after CD5 or CD7-Directed Chimeric Antigen Receptor T-Cell Therapy**

*Yuechen Luo, Haixiao Zhang, Kaiting Tang, Yiming Wang, Huajiang Dong, Wei Qi, Lingling Shan, Yue Tan, Liping Zhao, Jun Shi, Erlie Jiang*, Jing Pan*, Xiaoming Feng**

**This PDF file includes:**

Supplementary Methods

Figure S1 to S15

# Supplementary Methods

## *Single-cell V(D)J sequencing analysis*

BCR diversity was measured by Shannon and Inv.Simpson score. Shannon's entropy was calculated using the following formula: -∑(clonotype frequency * loge(clonotype frequency)). The Inverse Simpson index was calculated using the following formula: 1 / ∑(clonotype frequency * clonotype frequency). Both indices were derived using the clonalDiversity function.

Immunoglobulin heavy-chain (IGH) isotypes were classified based on the constant (C) region gene annotation: clonotypes containing IGHA1 or IGHA2 genes were defined as IgA; clonotypes containing IGHD were defined as IgD; clonotypes containing IGHG1, IGHG2, IGHG3, or IGHG4 genes were defined as IgG; clonotypes containing IGHM were defined IgM; clonotypes containing IGHE were defined IgE, however, these IgE clonotypes were only detected in the 7CAR group (samples S22 and S25) and were all Singleton (abundance = 1). Consequently, IgE clonotypes were not included in the results of group-level analyses due to their rarity. For statistical analysis, each clonotype

was further categorized by abundance: Singleton (abundance = 1) and Clonal (abundance > 1).

We annotated the CDR3 sequences of clonotypes using the immunarch v1.0.0 package (https://immunarch.com), utilizing existing TCR immune receptor databases, specifically VDJdb (https://vdjdb.cdr3.net) and McPAS-TCR (http://friedmanlab.weizmann.ac.il/McPAS-TCR). Initially, we extracted CDR3 amino acid sequences from both databases and merged the entries based on species or pathology, ensuring uniformity in category names to maintain consistency. After merging, redundant information was removed to guarantee the uniqueness of each entry. Due to the databases only containing individual CDR3 sequences from either the alpha or beta chain without paired sequence information, we performed stringent matches on single-chain CDR3 sequences.[1,2] Of the 120,786 records in these databases, approximately 36% correspond to α chains and 64% to β chains. No amino acid mismatches were allowed during this matching process to ensure the accuracy of the results. 10× VDJ files were processed, and pathogen-associated TCRs were identified

using the Immunarch package in R, as previously reported.[3,4] Based on the CDR3 sequence match results and the pathogen information recorded in the databases, we putatively defined TCRs associated with the following pathogens as virus-associated TCRs: HIV, CMV, SARS-CoV-2, EBV, HTLV-1, Influenza, HCV, HHV, YFV, HSV-2, HPV, MCPyV, Adenovirus, DENV, HCoV-HKU1, Hepatitis_E_virus_infection_cHEV, Human_herpes_virus_1. Similarly, TCRs associated with M.tuberculosis, E.Coli, SaccharomycesCerevisiae, PseudomonasFluorescens, PseudomonasAeruginosa, and StreptomycesKanamyceticus were defined as bacteria-associated TCRs.

We collected and analyzed the overlap of CDR3 sequences between samples and the merged database, weighting each overlapping CDR3 by its frequency within the sample repertoire to assess its representation.

For each cell type, the clonotypes present were determined.

For each sample, we initially grouped nucleotide sequences based on shared heavy chain V and J genes as well as CDR3 sequence lengths. Using SHazaM v1.2.0, we applied the findThreshold() function with default settings to establish a normalized

Hamming distance threshold for the CDR3 sequence repertoire, defining sequences exceeding this threshold as separate clones. Further refinement involved subdividing clones based on variations in light chain V and J genes or differences in CDR3 sequence lengths. Clonal germline consensus sequences for V and J segments were reconstructed using the createGermlines() function in dowser v2.3. The observedMutations() function in SHazaM calculated somatic hypermutation by assessing the total number and frequency of non-ambiguous nucleotide mismatches relative to the V and J genes within each cell's clonal germline sequence.[5]

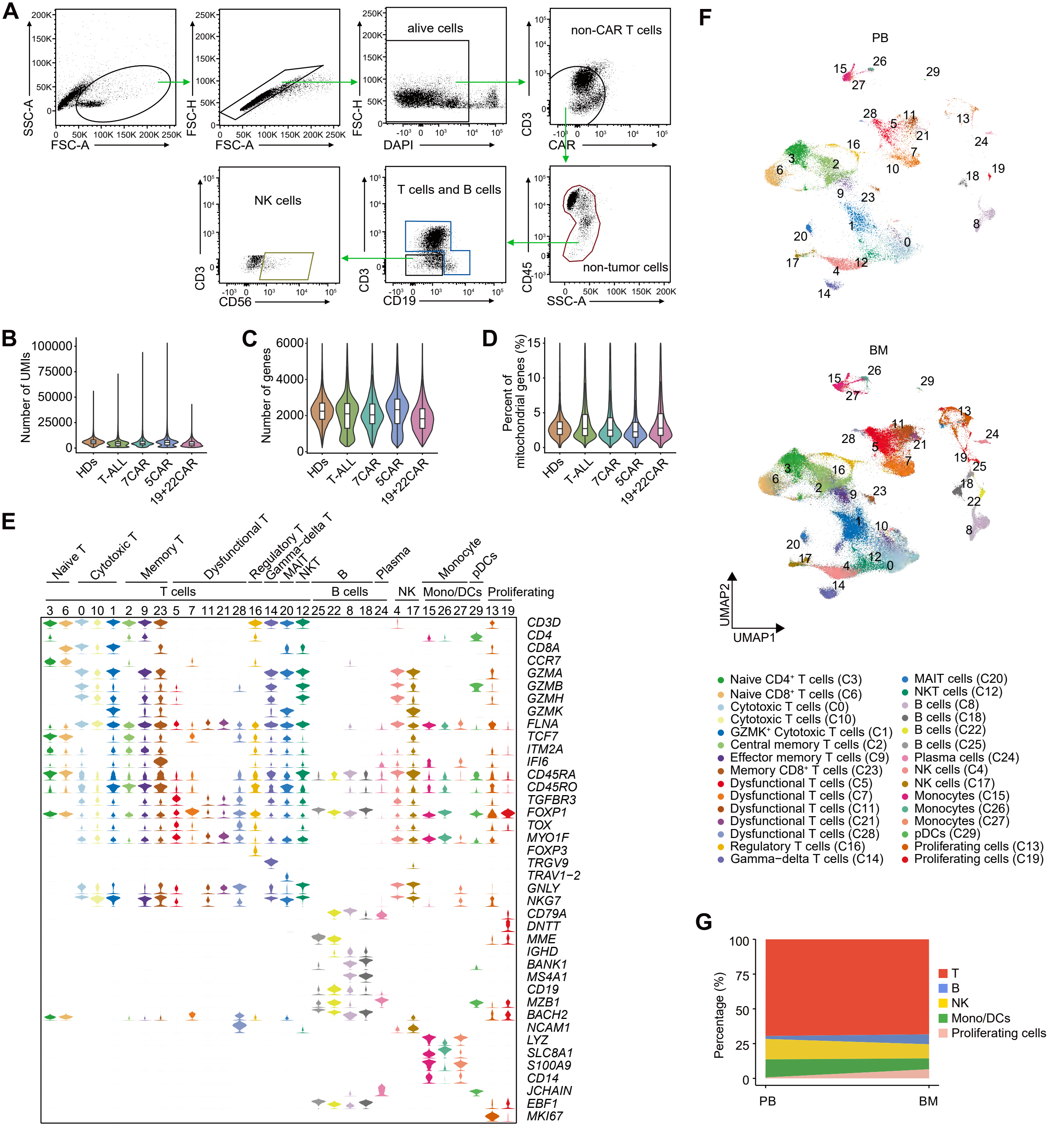
A
SSC-A
FSC-A
FSC-H
FSC-A
alive cells
FSC-H
DAPI
non-CAR T cells
CD3
CAR
NK cells
CD3
CD56
T cells and B cells
CD3
CD19
non-tumor cells
CD45
SSC-A
B
Number of UMIs
HDs
T-ALL
7CAR
5CAR
19+22CAR
C
Number of genes
D
Percent of mitochondrial genes (%)
E
Naive T
Cytotoxic T
Memory T
Dysfunctional T
Regulatory T
Gamma-delta T
MAIT
NKT
B
Plasma
Monocyte
pDCs
T cells
B cells
NK
Mono/DCs
Proliferating
3 6 0 10 1 2 9 23 5 7 11 21 28 16 14 20 12 25 22 8 18 24 4 17 15 26 27 29 13 19
CD3D
CD4
CD8A
CCR7
GZMA
GZMB
GZMH
GZMK
FLNA
TCF7
ITM2A
IFI6
CD45RA
CD45RO
TGFBR3
FOXP1
TOX
MYO1F
FOXP3
TRGV9
TRAV1−2
GNLY
NKG7
CD79A
DNTT
MME
IGHD
BANK1
MS4A1
CD19
MZB1
BACH2
NCAM1
LYZ
SLC8A1
S100A9
CD14
JCHAIN
EBF1
MKI67
F
PB
BM
UMAP2
UMAP1
Naive CD4⁺ T cells (C3)
Naive CD8⁺ T cells (C6)
Cytotoxic T cells (C0)
Cytotoxic T cells (C10)
GZMK⁺ Cytotoxic T cells (C1)
Central memory T cells (C2)
Effector memory T cells (C9)
Memory CD8⁺ T cells (C23)
Dysfunctional T cells (C5)
Dysfunctional T cells (C7)
Dysfunctional T cells (C11)
Dysfunctional T cells (C21)
Dysfunctional T cells (C28)
Regulatory T cells (C16)
Gamma−delta T cells (C14)
MAIT cells (C20)
NKT cells (C12)
B cells (C8)
B cells (C18)
B cells (C22)
B cells (C25)
Plasma cells (C24)
NK cells (C4)
NK cells (C17)
Monocytes (C15)
Monocytes (C26)
Monocytes (C27)
pDCs (C29)
Proliferating cells (C13)
Proliferating cells (C19)
G
Percentage (%)
PB
BM
T
B
NK
Mono/DCs
Proliferating cells

**Figure S1. Basic characteristics of the integrated dataset.**

(**A**) Enrichment gating strategy of human non-CAR non-tumor DAPI$^-$CD45$^+$ cells or DAPI$^-$CD45$^+$CD3$^+$ normal T cells, DAPI$^-$CD45$^+$CD19$^+$ normal B cells, and DAPI$^-$CD45$^+$CD56$^+$ NK cells. Tumor cells and CAR T cells were excluded.

(**B to D**) Distribution of UMI counts per cell (B), gene counts per cell (C), and percentage of mitochondrial genes per cell (D) detected for cells in different groups. Boxplots show median ± IQR, whiskers indicate full range.

(**E**) Violin plots showing marker genes in each cluster.

(**F**) UMAP plot of single-cell transcriptomes of cells from PB and BM samples, colored by clusters.

(**G**) Area plot of the proportion of cell types from PB (n = 3) and BM (n = 14) samples.

BM, bone marrow; CAR, chimeric antigen receptor; DCs, dendritic cells; HDs, healthy donors; MAIT, mucosal-associated invariant T cell; Mono, monocyte; NK, natural killer cell; NKT: natural killer T cell; PB, peripheral blood; pDCs, plasmacytoid dendritic cells; T-ALL, T-cell acute lymphoblastic leukemia; UMI, unique molecular identifier.

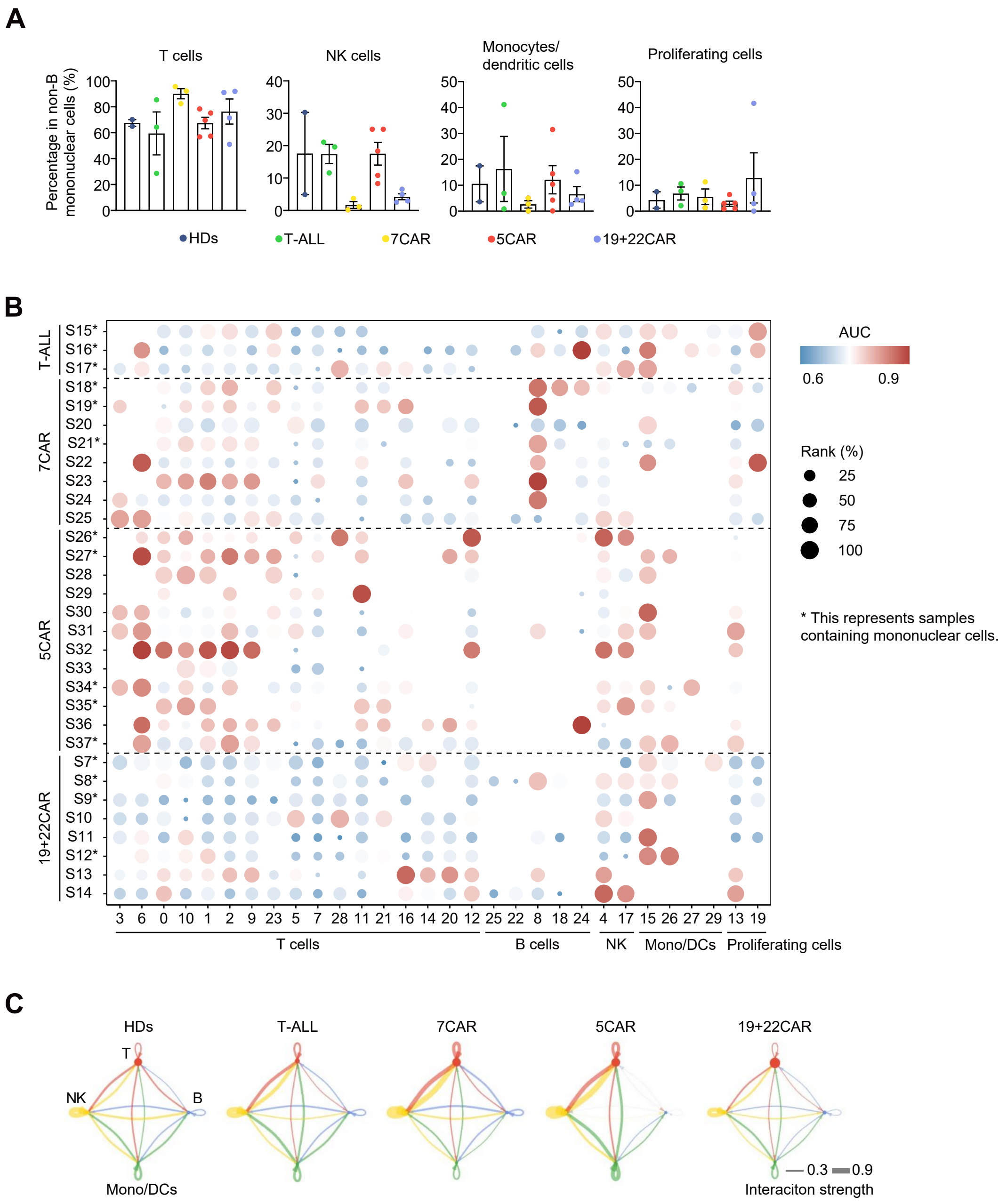
A
T cells
NK cells
Monocytes/
dendritic cells
Proliferating cells
Percentage in non-B
mononuclear cells (%)
HDs
T-ALL
7CAR
5CAR
19+22CAR
B
AUC
0.6
0.9
Rank (%)
25
50
75
100
* This represents samples containing mononuclear cells.
T-ALL
7CAR
5CAR
19+22CAR
T cells
B cells
NK
Mono/DCs
Proliferating cells
C
HDs
T-ALL
7CAR
5CAR
19+22CAR
T
NK
B
Mono/DCs
0.3
0.9
Interaciton strength

**Figure S2. Single-cell sequencing reveals immunodisturbance following different CAR-T treatments.**

(A) The proportion of each cell type in non-B mononuclear cells. Only samples containing mononuclear cells were used. Data are presented as means ± SEMs, n = 2 in HDs, n = 3 in T-ALL, n = 3 in 7CAR, n = 5 in 5CAR, n = 4 in 19+22CAR group.

(B) Prioritization of the most affected cell types in different conditions relative to healthy donors (HDs) condition by ranking Augur Cell Type Prioritization scores. Samples containing either mononuclear cells or lymphocytes (T, B and NK cells) were used. *This represents samples containing mononuclear cells. The rest samples contains lymphocytes (T, B, and NK cells).

(C) Circle plot showing inferred cell-cell communications. Samples containing either mononuclear cells or lymphocytes (T, B and NK cells) were used.

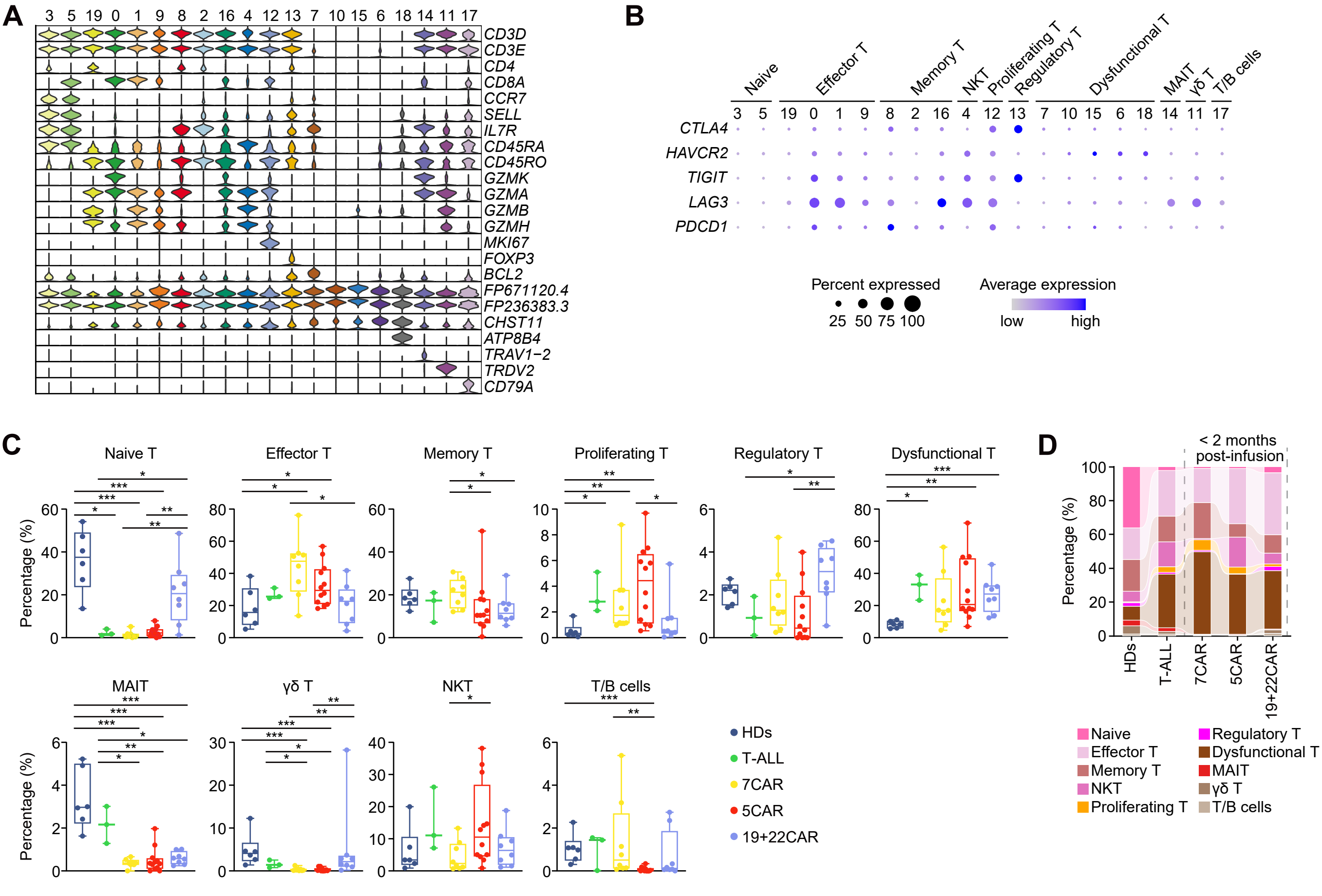
A
3 5 19 0 1 9 8 2 16 4 12 13 7 10 15 6 18 14 11 17
CD3D
CD3E
CD4
CD8A
CCR7
SELL
IL7R
CD45RA
CD45RO
GZMK
GZMA
GZMB
GZMH
MKI67
FOXP3
BCL2
FP671120.4
FP236383.3
CHST11
ATP8B4
TRAV1−2
TRDV2
CD79A
B
Naive
Effector T
Memory T
NKT
Proliferating T
Regulatory T
Dysfunctional T
MAIT
γδ T
T/B cells
3 5 19 0 1 9 8 2 16 4 12 13 7 10 15 6 18 14 11 17
CTLA4
HAVCR2
TIGIT
LAG3
PDCD1
Percent expressed
25 50 75 100
Average expression
low
high
C
Naive T
Effector T
Memory T
Proliferating T
Regulatory T
Dysfunctional T
MAIT
γδ T
NKT
T/B cells
Percentage (%)
HDs
T-ALL
7CAR
5CAR
19+22CAR
D
< 2 months post-infusion
Percentage (%)
HDs
T-ALL
7CAR
5CAR
19+22CAR
Naive
Effector T
Memory T
NKT
Proliferating T
Regulatory T
Dysfunctional T
MAIT
γδ T
T/B cells

**Figure S3. scRNA-seq reveals T-cell heterogeneity following various CAR T-cell therapies.**

(**A**) Violin plots showing marker genes in each T-cell cluster.

(**B**) The expression of representative exhaustion markers is shown for each cluster. The dot size represents the percentage of cells expressing the indicated genes, and the dot color shows the averaged expression level. T/B cells: expression of both T and B cell marker genes (*CD3E*, *CD79A*).

(**C**) Comparison of the percentage of specific T-cell populations in different groups across all samples. Boxplots show median ± IQR, whiskers indicate full range, n = 6 in HDs, n = 3 in T-ALL, n = 8 in 7CAR, n = 12 in 5CAR, n = 8 in 19+22CAR group. Dots represent individual samples. *P* value by two-sided unpaired Mann-Whitney test.

(**D**) Relative percentages of T-cell populations in all healthy donors, CAR-naïve T-ALL patients, and patients within 2 months of CAR T-cell therapies.

*$p < 0.05$, **$p < 0.01$, ***$p < 0.001$. CAR, chimeric antigen receptor; HDs, healthy donors; MAIT, mucosal-associated invariant T cell; NKT: natural killer T cell; T-ALL, T-cell acute lymphoblastic leukemia; Treg, regulatory T cell.

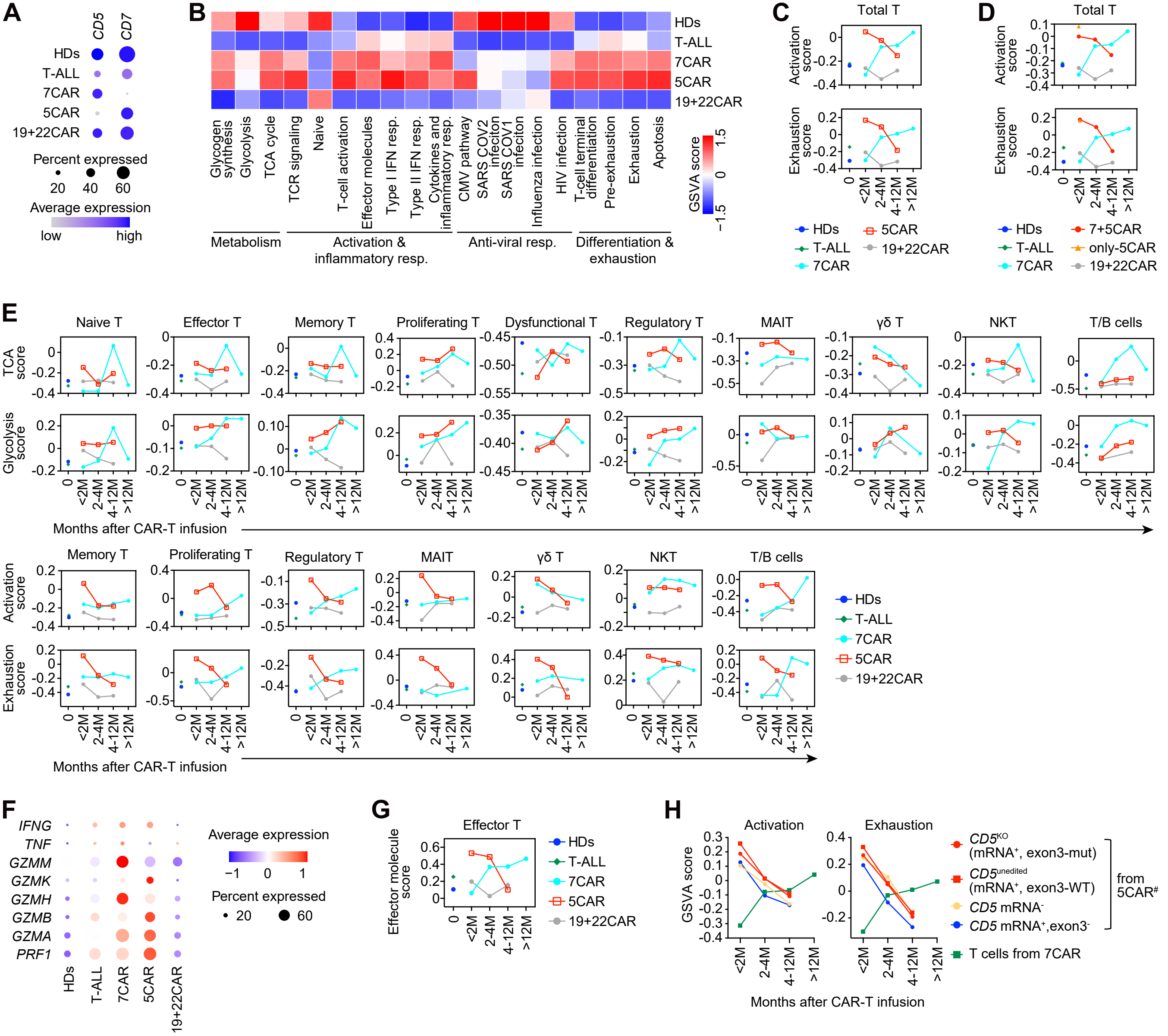
A
CD5
CD7
HDs
T-ALL
7CAR
5CAR
19+22CAR
Percent expressed
20 40 60
Average expression
low high
B
HDs
T-ALL
7CAR
5CAR
19+22CAR
Glycogen synthesis
Glycolysis
TCA cycle
TCR signaling
Naive
T-cell activation
Effector molecules
Type I IFN resp.
Type II IFN resp.
Cytokines and inflammatory resp.
CMV pathway
SARS COV2 infeciton
SARS COV1 infeciton
Influenza infection
HIV infection
T-cell terminal differentiation
Pre-exhaustion
Exhaustion
Apoptosis
GSVA score
Metabolism
Activation & inflammatory resp.
Anti-viral resp.
Differentiation & exhaustion
C
Total T
Activation score
Exhaustion score
HDs
T-ALL
7CAR
5CAR
19+22CAR
D
Total T
Activation score
Exhaustion score
HDs
T-ALL
7CAR
7+5CAR
only-5CAR
19+22CAR
E
Naive T
Effector T
Memory T
Proliferating T
Dysfunctional T
Regulatory T
MAIT
γδ T
NKT
T/B cells
TCA score
Glycolysis score
0 <2M 2-4M 4-12M >12M
Months after CAR-T infusion
Memory T
Proliferating T
Regulatory T
MAIT
γδ T
NKT
T/B cells
Activation score
Exhaustion score
HDs
T-ALL
7CAR
5CAR
19+22CAR
Months after CAR-T infusion
F
IFNG
TNF
GZMM
GZMK
GZMH
GZMB
GZMA
PRF1
HDs
T-ALL
7CAR
5CAR
19+22CAR
Average expression
Percent expressed
20 60
G
Effector T
Effector molecule score
HDs
T-ALL
7CAR
5CAR
19+22CAR
H
Activation
Exhaustion
GSVA score
CD5KO (mRNA+, exon3-mut)
CD5unedited (mRNA+, exon3-WT)
CD5 mRNA-
CD5 mRNA+,exon3-
from 5CAR#
T cells from 7CAR
Months after CAR-T infusion

**Figure S4. scRNA-seq reveals alterations in T-cell function following various CAR T-cell therapies.**

(**A**) Dot plot showing the expression of CD7 and CD5 in T cells in different groups. The dot size represents the percentage of cells expressing the indicated genes, and the dot color shows the averaged expression level.

(**B**) Heatmap showing GSVA enrichment score of genes associated with T-cell metabolism, activation, inflammatory response, anti-viral response, differentiation and exhaustion in all patients and healthy donors.

(**C and D**) Line plots showing the GSVA enrichment score of activation and exhaustion at different time points in total T cells among distinct groups including the 5CAR group (**C**) or the 7+5CAR and only-5CAR groups (**D**).

(**E**) Line plots showing the GSVA enrichment score of TCA cycle, glycolysis, activation, and exhaustion at different time points in each T-cell type among distinct groups.

(**F**) Dot plot showing the expression of effector molecules in T cells in different groups. The dot size represents the percentage of cells expressing the indicated genes, and the dot color shows the averaged expression level.

(**G**) Line plots representing the GSVA enrichment score of effector molecules at different time points in effector T cells among distinct groups.

(**H**) GSVA enrichment score of activation and exhaustion across different editing states of the *CD5* gene in T cells from 5CAR group and 7CAR group at different time points among distinct groups. $^{\#}$ Indicates that T cells were from samples that were confirmed to be CD5 protein-negative by flow cytometry. *CD5* mRNA$^{+}$ and exon3$^{-}$ indicates *CD5* mRNA$^{+}$ and exon3-undetected T cells, and it was not clear whether exon3 was edited by gRNA.

CAR, chimeric antigen receptor; GSVA, gene set variation analysis; HDs, healthy donors; HIV, human immunodeficiency virus; IFN, interferon; resp., response; M, month; MAIT, mucosal-associated invariant T cell; NKT: natural killer T cell; SARS-CoV-2, severe acute respiratory syndrome coronavirus 2; T-ALL, T-cell acute lymphoblastic leukemia; TCA, tricarboxylic acid; TCR, T-cell receptor; Treg, regulatory T cell.

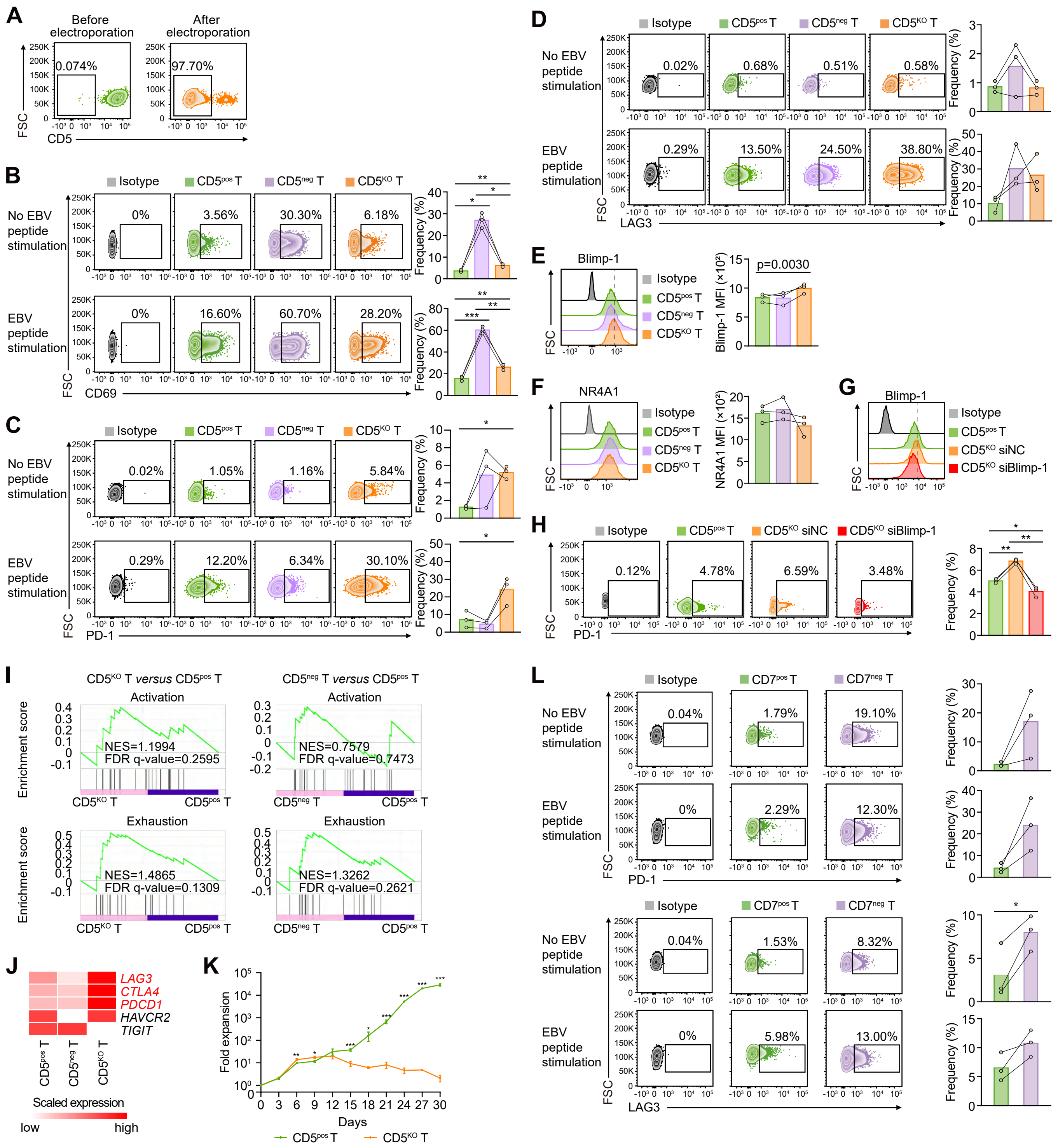
A
Before electroporation
After electroporation
0.074%
97.70%
FSC
CD5
B
Isotype
CD5pos T
CD5neg T
CD5KO T
No EBV peptide stimulation
EBV peptide stimulation
0%
3.56%
30.30%
6.18%
16.60%
60.70%
28.20%
CD69
Frequency (%)
C
0.02%
1.05%
1.16%
5.84%
0.29%
12.20%
6.34%
30.10%
PD-1
D
0.68%
0.51%
0.58%
13.50%
24.50%
38.80%
LAG3
E
Blimp-1
Blimp-1 MFI (×10²)
p=0.0030
F
NR4A1
NR4A1 MFI (×10²)
G
CD5KO siNC
CD5KO siBlimp-1
H
0.12%
4.78%
6.59%
3.48%
I
CD5KO T versus CD5pos T
CD5neg T versus CD5pos T
Activation
Exhaustion
Enrichment score
NES=1.1994
FDR q-value=0.2595
NES=0.7579
FDR q-value=0.7473
NES=1.4865
FDR q-value=0.1309
NES=1.3262
FDR q-value=0.2621
J
LAG3
CTLA4
PDCD1
HAVCR2
TIGIT
Scaled expression
low
high
K
Fold expansion
Days
L
CD7pos T
CD7neg T
0.04%
1.79%
19.10%
0%
2.29%
12.30%
1.53%
8.32%
5.98%
13.00%

**Figure S5.** ***In vitro*** **assays revealed the distinct activation and exhaustion functions of $CD5^{pos}$, $CD5^{neg}$ and $CD5^{KO}$ T cells, as well as $CD7^{pos}$ and $CD7^{neg}$ T cells.**

(**A**) CD5 expression of T cells before and after electroporation as measured by flow cytometry.

(**B**) CD69 expression in $CD5^{pos}$, $CD5^{neg}$, and $CD5^{KO}$ T cells, with or without two days of EBV-peptide stimulation, measured by flow cytometry. n = 3.

(**C**) PD-1 expression in $CD5^{pos}$, $CD5^{neg}$, and $CD5^{KO}$ T cells, with or without two days of EBV-peptide stimulation, measured by flow cytometry. n = 3.

(**D**) Expression of LAG3 in $CD5^{pos}$, $CD5^{neg}$, and $CD5^{KO}$ T cells, with or without two days of EBV-peptide stimulation, as measured by flow cytometry. n = 3. PD-1 (C) and LAG-3 (D) were detected using the same fluorochrome channel, so a single isotype control was used for both, with or without EBV-peptide stimulation.

(**E**) Blimp-1 expression in $CD5^{pos}$, $CD5^{neg}$, and $CD5^{KO}$ T cells, with two days of EBV-peptide stimulation, measured by flow cytometry. n = 3.

(**F**) Expression of NR4A1 in $CD5^{pos}$, $CD5^{neg}$, and $CD5^{KO}$ T cells, with EBV-peptide stimulation for 2 days, as measured by flow cytometry. n = 3.

(**G**) SiRNA targeting Blimp-1 (siBlimp-1) was used to disrupt the expression of Blimp-1 in $CD5^{KO}$ T cells with negative control siRNA as comparison.

(**H**) PD-1 expression in $CD5^{pos}$ T cells, and $CD5^{KO}$ T cells transfected with siNC or siBlimp-1, measured by flow cytometry. n = 3.

(**I**) GSEA results from running bulk RNA sequencing data of $CD5^{KO}$ T versus $CD5^{pos}$ T cells, or $CD5^{neg}$ versus $CD5^{pos}$ T cells, with EBV-peptide stimulation for 2 days. FDR q values and NES were calculated using GSEA software. n = 1.

(**J**) Expression of exhaustion associated genes in $CD5^{pos}$, $CD5^{neg}$, and $CD5^{KO}$ T cells with

EBV-peptide stimulation for 2 days, bulk RNA sequencing data. n = 1.

(**K**) $CD5^{pos}$ and $CD5^{KO}$ T cell expansion *in vitro*. Data presented as mean ± SD. Three technical replicates. *P* value by unpaired t-test.

(**L**) Expression of exhaustion associated genes in $CD7^{pos}$ and $CD7^{neg}$ T cells, with or without EBV-peptide stimulation for 2 days, as measured by flow cytometry. n = 3, paired t-test. PD-1 and LAG-3 were detected using the same fluorochrome channel, so a single isotype control was used for both, with or without EBV-peptide stimulation.

$CD5^{pos}$, $CD5^{neg}$, $CD5^{KO}$, $CD7^{pos}$ and $CD7^{neg}$ T cells were isolated or generated from the healthy donors. The *p* values in panels (B-F) and (H) were calculated by one-way RM ANOVA analysis and subsequent Tukey's multiple comparisons test. $^*p < 0.05$, $^{**}p < 0.01$, $^{***}p < 0.001$. EBV, Epstein Barr virus; FDR, false discovery rate; GSEA, gene set enrichment analysis; NES, normalized enrichment score.

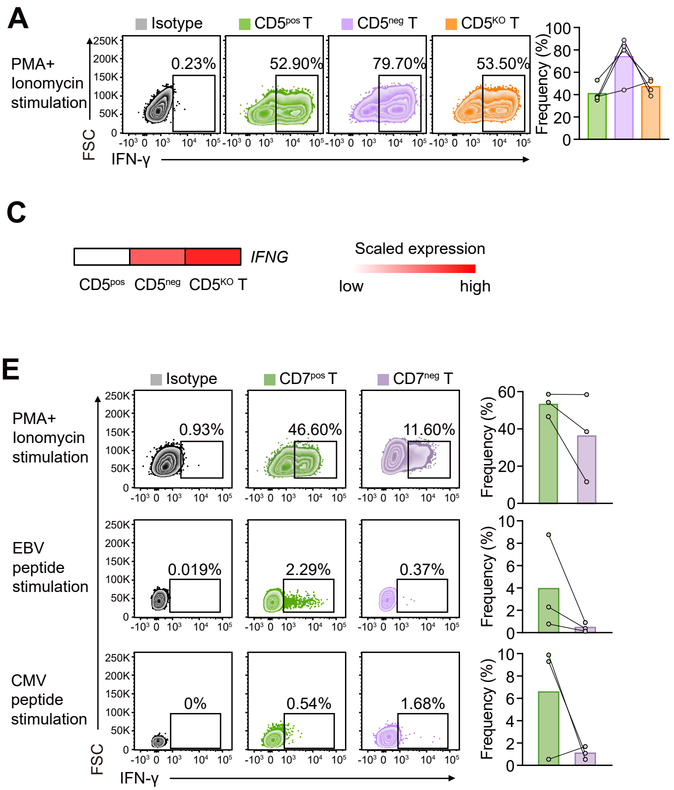
A
Isotype
CD5pos T
CD5neg T
CD5KO T
PMA+ Ionomycin stimulation
0.23%
52.90%
79.70%
53.50%
FSC
IFN-γ
Frequency (%)
B
EBV peptide stimulation
0%
1.86%
2.48%
0.27%
C
IFNG
CD5pos CD5neg CD5KO T
Scaled expression
low
high
D
CMV peptide stimulation
0.088%
0.34%
7.76%
0.073%
E
CD7pos T
CD7neg T
0.93%
46.60%
11.60%
0.019%
2.29%
0.37%
0%
0.54%
1.68%

**Figure S6.** ***In vitro*** **assays revealed the distinct IFN-γ expression of $CD5^{pos}$, $CD5^{neg}$ and $CD5^{KO}$ T cells, as well as $CD7^{pos}$ and $CD7^{neg}$ T cells.**

(**A** and **B**) IFN-γ expression in $CD5^{pos}$, $CD5^{neg}$, and $CD5^{KO}$ T cells, with PMA+ionomycin (**A**) or two days of EBV-peptide stimulation (**B**), measured by flow cytometry. n = 4.

(**C**) Expression of *IFNG* in $CD5^{pos}$, $CD5^{neg}$, and $CD5^{KO}$ T cells, with EBV-peptide stimulation for 2 days, bulk RNA sequencing data. n = 1.

(**D**) Expression of IFN-γ in $CD5^{pos}$, $CD5^{neg}$, and $CD5^{KO}$ T cells with CMV-peptide stimulation for 2 days, as measured by flow cytometry. n = 3.

(**E**) Expression of IFN-γ in $CD7^{pos}$ and $CD7^{neg}$ T cells with PMA+ionomycin, EBV-peptide, or CMV-peptide stimulation stimulation for 2 days, as measured by flow cytometry. n = 3, paired t-test.

$CD5^{pos}$, $CD5^{neg}$, $CD5^{KO}$, $CD7^{pos}$ and $CD7^{neg}$ T cells were isolated or generated from the healthy donors. The $p$ values in panels (A-B) and (D) were calculated by one-way RM ANOVA analysis and subsequent Tukey's multiple comparisons test. $^{*}p < 0.05$. IFN, interferon.

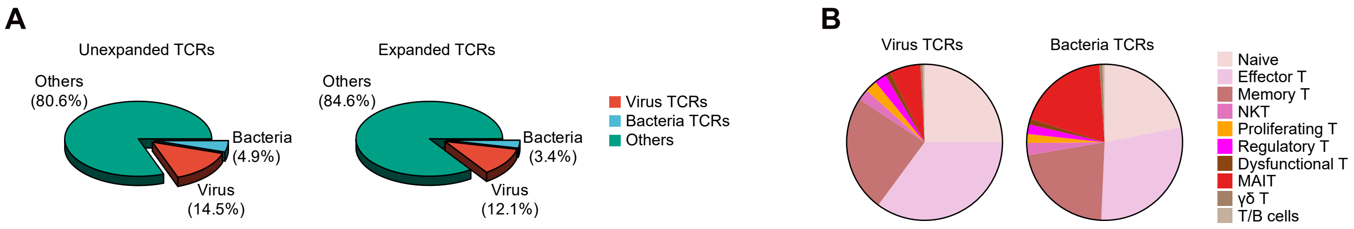
A
Unexpanded TCRs
Others
(80.6%)
Bacteria
(4.9%)
Virus
(14.5%)
Expanded TCRs
Others
(84.6%)
Bacteria
(3.4%)
Virus
(12.1%)
Virus TCRs
Bacteria TCRs
Others
B
Virus TCRs
Bacteria TCRs
Naive
Effector T
Memory T
NKT
Proliferating T
Regulatory T
Dysfunctional T
MAIT
γδ T
T/B cells

**Figure S7. scTCR-seq reveals alterations in T cells.**

(**A**) Percentage of different TCRs in unexpanded or expanded T cells.

(**B**) Proportion of each cell type in the virus- or bacteria-associated TCRs.

CAR, chimeric antigen receptor; MAIT, mucosal-associated invariant T cell; NKT: natural killer T cell; TCR, T-cell receptor.

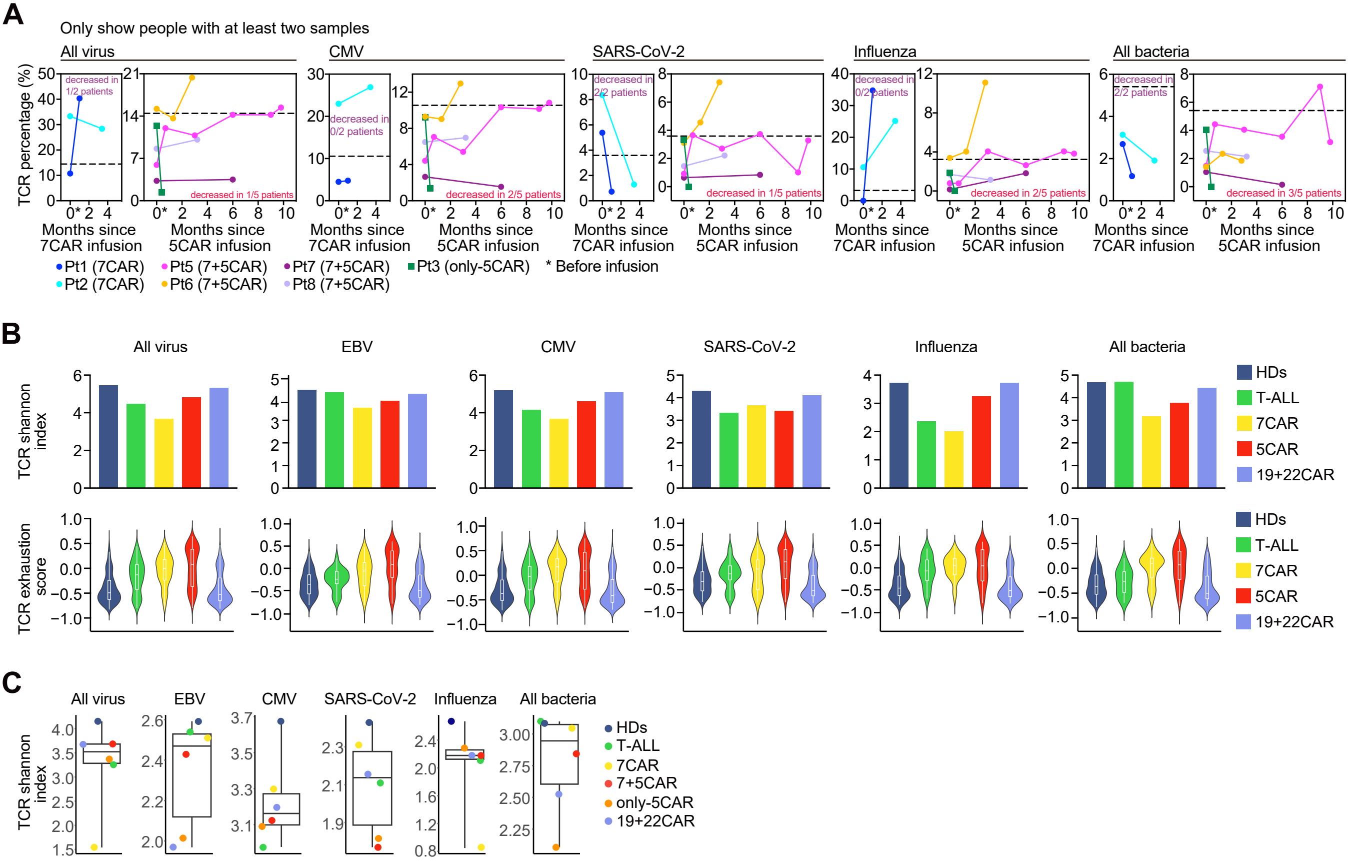
A
Only show people with at least two samples
All virus
CMV
SARS-CoV-2
Influenza
All bacteria
TCR percentage (%)
decreased in 1/2 patients
decreased in 1/5 patients
decreased in 0/2 patients
decreased in 2/5 patients
decreased in 2/2 patients
decreased in 1/5 patients
decreased in 0/2 patients
decreased in 2/5 patients
decreased in 2/2 patients
decreased in 3/5 patients
Months since 7CAR infusion
Months since 5CAR infusion
Pt1 (7CAR)
Pt2 (7CAR)
Pt5 (7+5CAR)
Pt6 (7+5CAR)
Pt7 (7+5CAR)
Pt8 (7+5CAR)
Pt3 (only-5CAR)
* Before infusion
B
All virus
EBV
CMV
SARS-CoV-2
Influenza
All bacteria
TCR shannon index
TCR exhaustion score
HDs
T-ALL
7CAR
5CAR
19+22CAR
C
All virus
EBV
CMV
SARS-CoV-2
Influenza
All bacteria
TCR shannon index
HDs
T-ALL
7CAR
7+5CAR
only-5CAR
19+22CAR

**Figure S8. Pathogen associated TCRs following various CAR T-cell therapies.**

(**A**) Percentage of indicated associated TCRs before and after CD7 or CD5 CAR T-cell treatment in the same individual patient. Different colors indicate different patients.

(**B**) Diversity (top) and exhaustion score (bottom) of indicated associated TCRs from all patients and healthy donors.

(**C**) TCR diversity of indicated associated TCRs from samples within 2 months in each group. Boxplots show median ± IQR, whiskers indicate full range.

CAR, chimeric antigen receptor; CMV, cytomegalovirus; EBV, Epstein Barr virus; HDs, healthy donors; SARS-CoV-2, severe acute respiratory syndrome coronavirus 2; T-ALL, T-cell acute lymphoblastic leukemia; TCR, T-cell receptor.

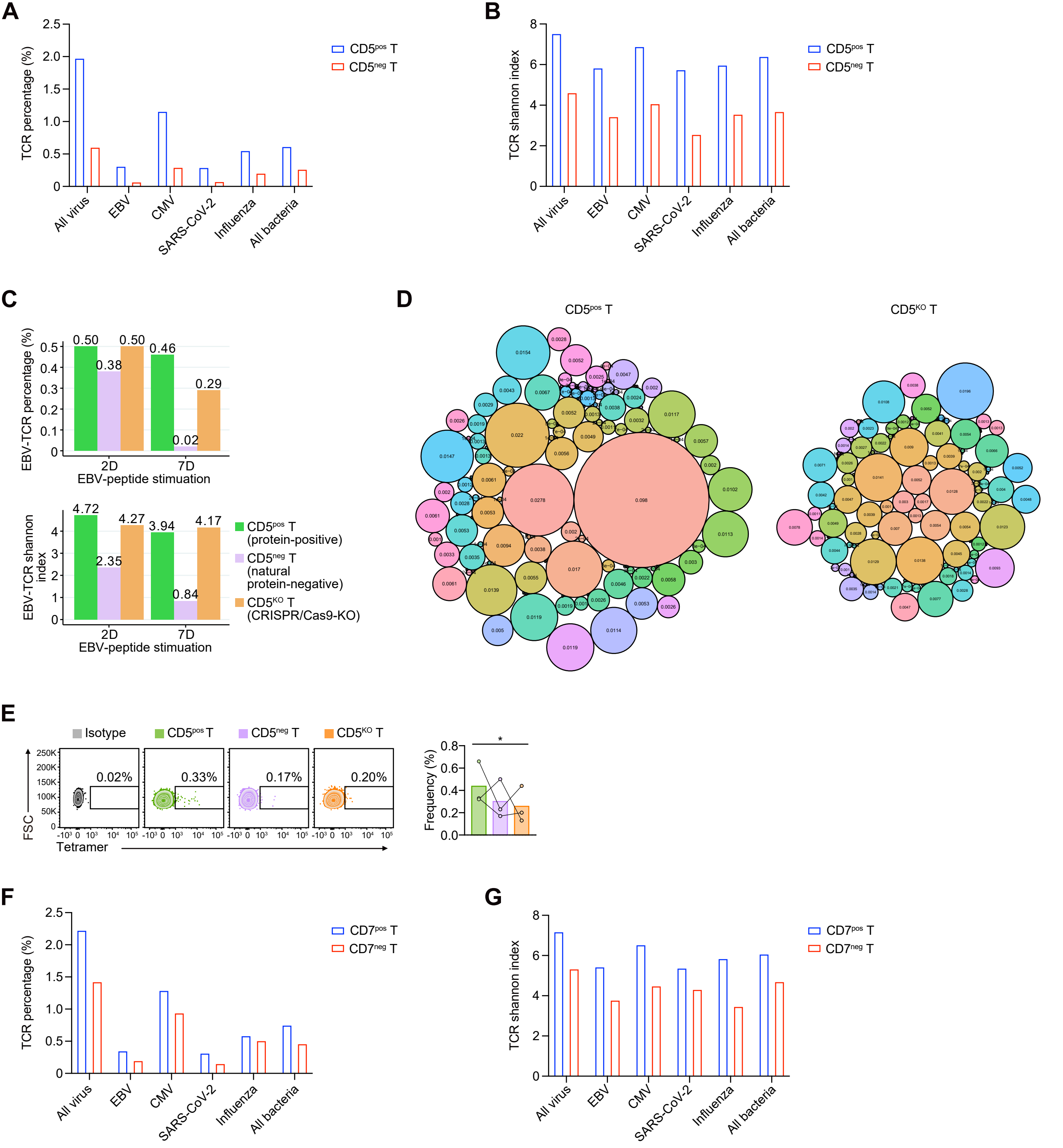
A
TCR percentage (%)
2.5
2.0
1.5
1.0
0.5
0
All virus
EBV
CMV
SARS-CoV-2
Influenza
All bacteria
CD5pos T
CD5neg T
B
TCR shannon index
8
6
4
2
0
C
EBV-TCR percentage (%)
0.50
0.38
0.50
0.46
0.02
0.29
2D
7D
EBV-peptide stimulation
EBV-TCR shannon index
4.72
2.35
4.27
3.94
0.84
4.17
CD5pos T (protein-positive)
CD5neg T (natural protein-negative)
CD5KO T (CRISPR/Cas9-KO)
D
CD5pos T
CD5KO T
E
Isotype
CD5pos T
CD5neg T
CD5KO T
0.02%
0.33%
0.17%
0.20%
FSC
Tetramer
250K
200K
150K
100K
50K
0
Frequency (%)
0.8
0.6
0.4
0.2
0.0
*
F
CD7pos T
CD7neg T
G

**Figure S9. Bulk TCR-seq reveals distinct characteristics of CD5$^{pos}$, CD5$^{neg}$, and CD5$^{KO}$ T cells.**

(**A and B**) Percentage (**A**) and diversity (**B**) of indicated TCRs from sorted CD5$^{pos}$ and CD5$^{neg}$ T cells from healthy donors (n = 1). These cells were not treated in any way after sorting.

(**C**) EBV-associated TCR percentages and diversity of CD5$^{pos}$, CD5$^{neg}$, and CD5$^{KO}$ T cells, after EBV-peptide stimulation for 2 or 7 days, determined by bulk TCR-seq. n = 1.

(**D**) Percentage of annotated EBV-associated clone in all detected TCRs. CD5$^{pos}$ and CD5$^{KO}$ T cells from healthy donors (n = 1) were co-cultured with DCs, under the stimulation of EBV-peptide for 7 days.

(**E**) EBV-specific tetramer$^{+}$CD8$^{+}$ T percentage in CD5$^{pos}$, CD5$^{neg}$, and CD5$^{KO}$ T cells, after EBV-peptide stimulation for 7 days, stained with EBV mix tetramer and analyzed by flow cytometry. A total of three donors were used as biological replicates in three independent experiments. n = 3, one-way RM ANOVA analysis and subsequent Tukey's multiple comparisons test.

(**F and G**) Percentage (**F**) and diversity (**G**) of indicated TCRs from sorted CD7$^{pos}$ and CD7$^{neg}$ T cells from healthy donors (n = 1). These cells were not treated in any way after sorting.

*$p < 0.05$. CMV, cytomegalovirus; EBV, Epstein Barr virus; SARS-CoV-2, severe acute respiratory syndrome coronavirus 2; TCR, T-cell receptor.

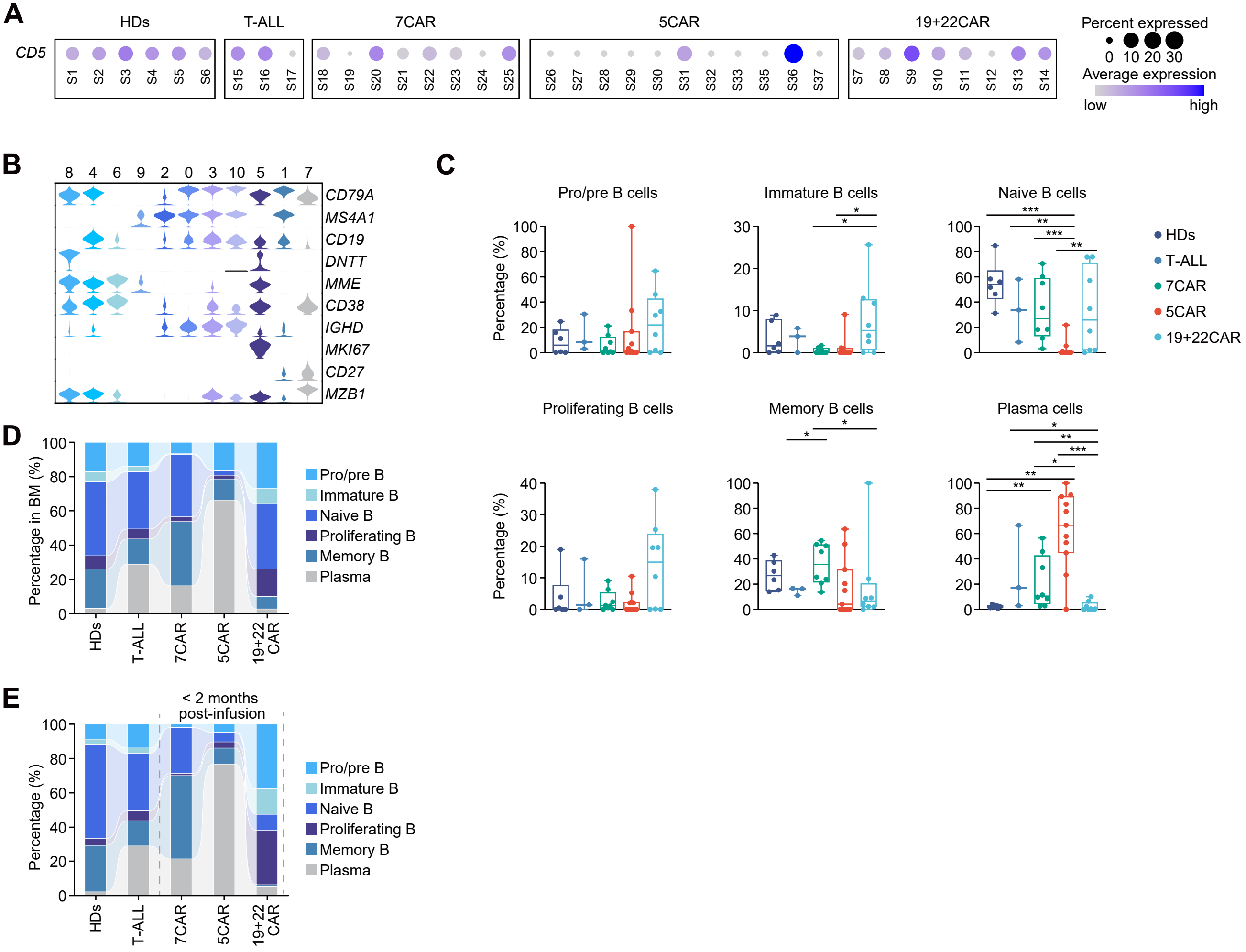
A
HDs
T-ALL
7CAR
5CAR
19+22CAR
CD5
S1 S2 S3 S4 S5 S6
S15 S16 S17
S18 S19 S20 S21 S22 S23 S24 S25
S26 S27 S28 S29 S30 S31 S32 S33 S35 S36 S37
S7 S8 S9 S10 S11 S12 S13 S14
Percent expressed
0 10 20 30
Average expression
low
high
B
8 4 6 9 2 0 3 10 5 1 7
CD79A
MS4A1
CD19
DNTT
MME
CD38
IGHD
MKI67
CD27
MZB1
C
Pro/pre B cells
Immature B cells
Naive B cells
Proliferating B cells
Memory B cells
Plasma cells
Percentage (%)
HDs
T-ALL
7CAR
5CAR
19+22CAR
D
Percentage in BM (%)
HDs
T-ALL
7CAR
5CAR
19+22 CAR
Pro/pre B
Immature B
Naive B
Proliferating B
Memory B
Plasma
E
< 2 months post-infusion
Percentage (%)
HDs
T-ALL
7CAR
5CAR
19+22 CAR
Pro/pre B
Immature B
Naive B
Proliferating B
Memory B
Plasma

**Figure S10. scRNA-seq reveals B-cell percentage changes after different CAR T-cell therapies.**

(**A**) Dot plot showing the expression of *CD5* in B cells of each sample in different groups. The dot size represents the percentage of cells expressing the indicated genes, and the dot color shows the averaged expression level. Sample 34, which contained only one B cell in Figure 1C, was excluded from B-cell subclustering.

(**B**) Violin plots showing marker genes in each B-cell cluster.

(**C**) Comparison of the percentage of specific B-cell populations in different groups across all samples. Boxplots show median ± IQR, whiskers indicate full range, n = 6 in HDs, n = 3 in T-ALL, n = 8 in 7CAR, n = 11 in 5CAR, n = 8 in 19+22CAR group. Dots represent individual samples. *P* value by two-sided unpaired Mann-Whitney test.

(**D**) Relative percentages of B-cell clusters in bone marrow in all patients and healthy donors.

(**E**) Relative percentages of B-cell clusters in all patients and healthy donors within 2 months of CAR T-cell therapies.

*$p < 0.05$, **$p < 0.01$, ***$p < 0.001$. CAR, chimeric antigen receptor; HDs, healthy donors; T-ALL, T-cell acute lymphoblastic leukemia.

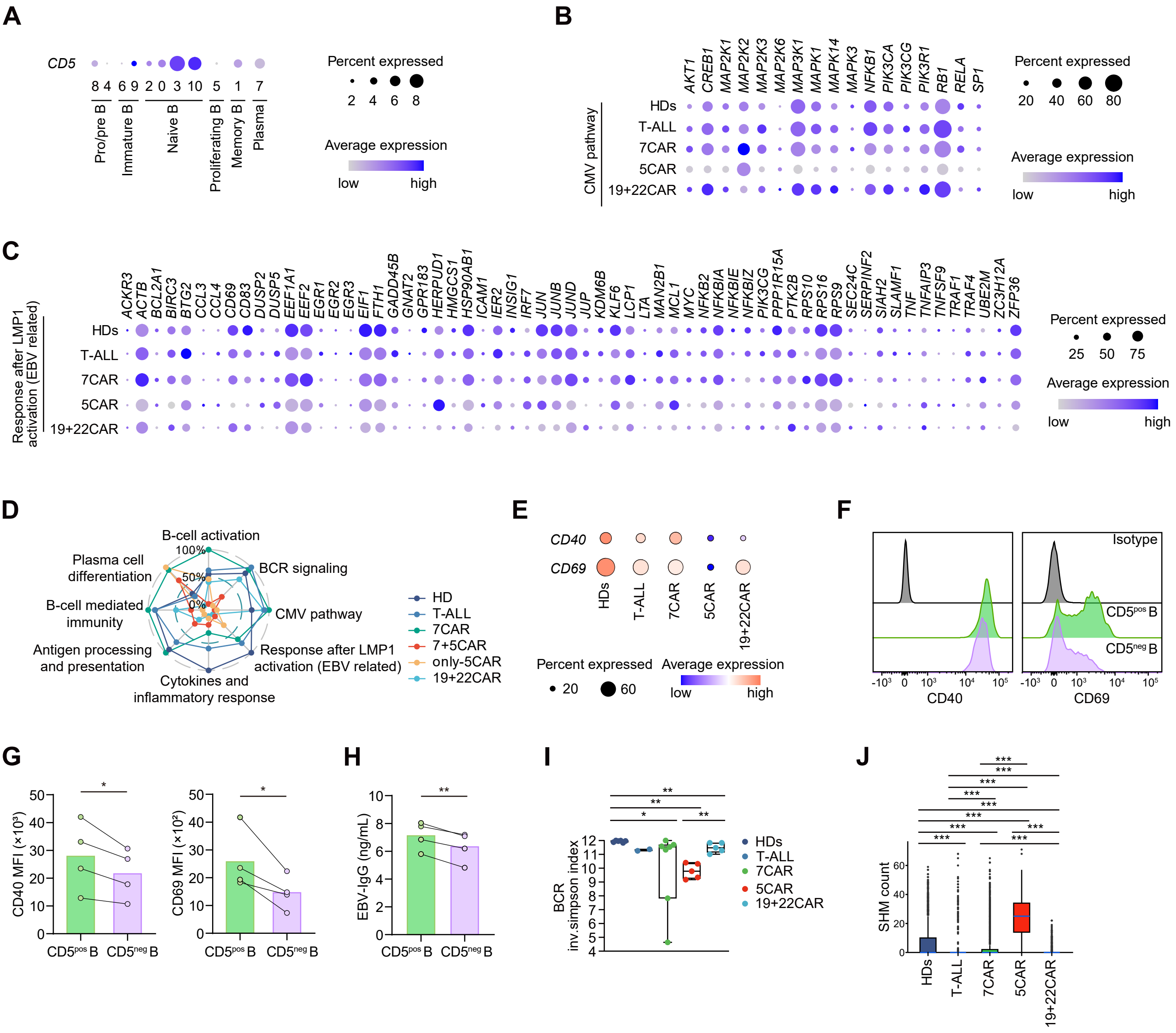

**Figure S11. scRNA-seq reveals B-cell function changes after different CAR T-cell therapies.**

(**A**) Dot plot showing the expression of *CD5* in different subpopulations. Dot size represents the percentage of cells expressing the indicated genes, and the dot color shows the averaged expression level.

(**B**) Dot plot showing the expression of CMV pathway genes in all B cells in each group. Dot size represents the percentage of cells expressing the indicated genes, and dot color shows the averaged level of expression.

(**C**) Dot plot showing the expression of genes related to response after LMP1 (EBV related) in all B cells in each group. Dot size represents the percentage of cells expressing the indicated genes, and dot color shows the averaged level of expression.

(**D**) Radar plot shows the GSVA enrichment score of B-cell characteristic pathways normalized by all scored cells in all healthy donors, CAR-naïve T-ALL patients, and patients within 2 months of CAR T-cell therapies, ranging from 0 to 100%.

(**E**) *CD69* and *CD40* expression.

(**F** and **G**) CD69 and CD40 expression in $CD5^{pos}$ and $CD5^{neg}$ B cells measured by flow cytometry. n = 4, paired t-test.

(**H**) EBV-specific IgG quantification in $CD5^{pos}$ and $CD5^{neg}$ B cells measured by ELISA. n = 4, paired t-test.

(**I**) BCR diversity. Boxplots show median ± IQR, whiskers indicate full range, n = 6 in HDs, n = 2 in T-ALL, n = 7 in 7CAR, n = 5 in 5CAR, n = 5 in 19+22CAR group. *P* value by two-sided unpaired Mann-Whitney test.

(**J**) Count of somatic hypermutation based on scBCR-seq. Boxplots show median ± IQR, whiskers indicate full range with excluding outliers. *P* value by two-sided unpaired Mann-Whitney test.

*$p < 0.05$, **$p < 0.01$, ***$p < 0.001$. BCR, B-cell receptor; CAR, chimeric antigen receptor; CMV, cytomegalovirus; EBV, Epstein Barr virus; HDs, healthy donors; LMP1, latent membrane protein 1; SHM, somatic hypermutation; T-ALL, T-cell acute lymphoblastic leukemia.

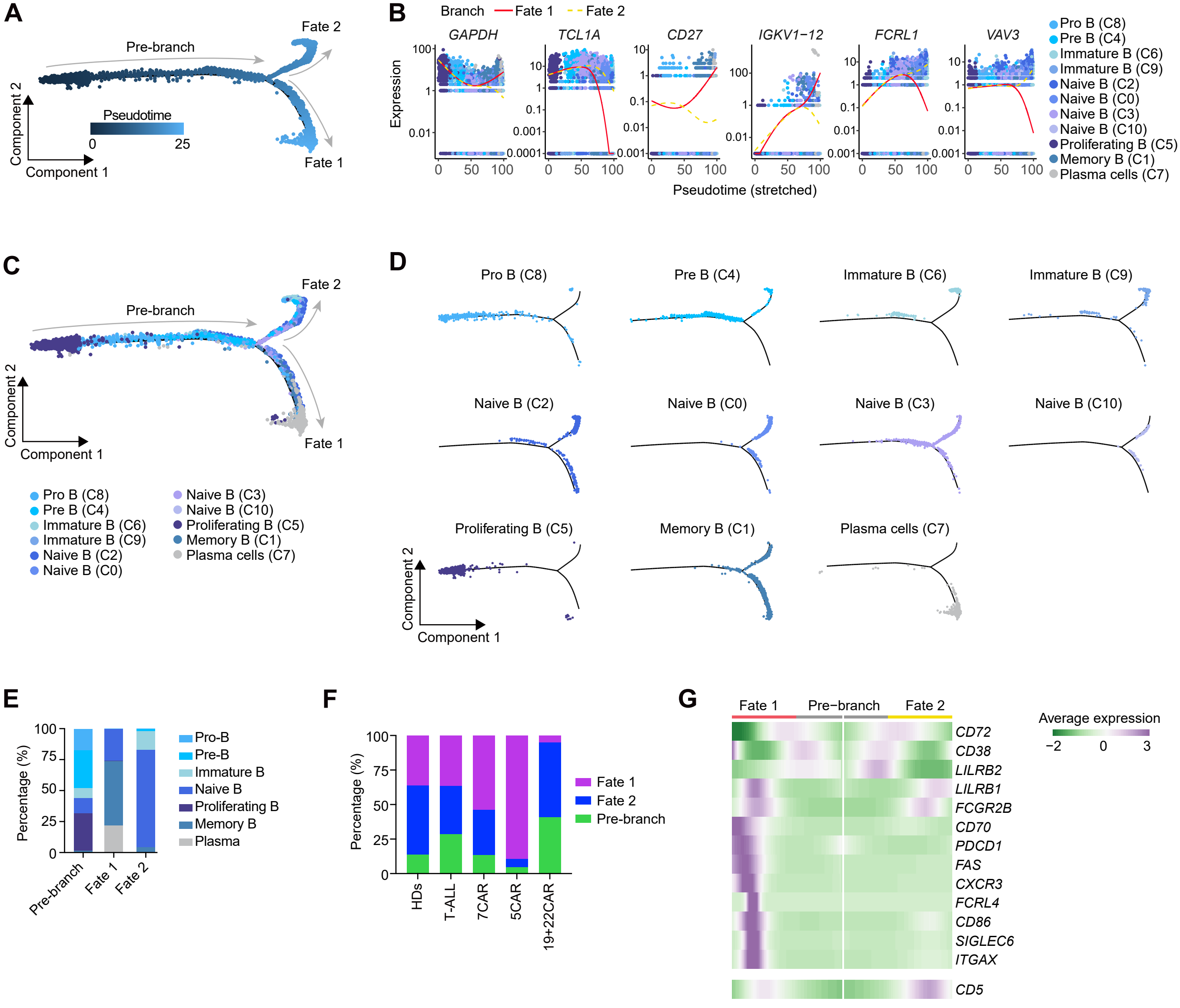

A
Pre-branch
Fate 2
Fate 1
Pseudotime
0
25
Component 2
Component 1
B
Branch
Fate 1
Fate 2
GAPDH
TCL1A
CD27
IGKV1−12
FCRL1
VAV3
Expression
Pseudotime (stretched)
Pro B (C8)
Pre B (C4)
Immature B (C6)
Immature B (C9)
Naive B (C2)
Naive B (C0)
Naive B (C3)
Naive B (C10)
Proliferating B (C5)
Memory B (C1)
Plasma cells (C7)
C
D
E
Percentage (%)
Pre-branch
Fate 1
Fate 2
Pro-B
Pre-B
Immature B
Naive B
Proliferating B
Memory B
Plasma
F
HDs
T-ALL
7CAR
5CAR
19+22CAR
G
Average expression
CD72
CD38
LILRB2
LILRB1
FCGR2B
CD70
PDCD1
FAS
CXCR3
FCRL4
CD86
SIGLEC6
ITGAX
CD5

**Figure S12. Pseudotime analysis of B cells.**

(**A**) Pseudotime trajectory of B cells. Dots represent individual cells.

(**B**) Representative marker genes for each branch. Dots represent individual cells.

(**C**) Pseudotime trajectory of B cells.

(**D**) Pseudotime trajectory of different B-cell clusters. Dots represent individual cells.

(**E**) Proportion of different B-cell types in each path.

(**F**) Proportions of different B-cell paths.

(**G**) Pseudotemporal gene-expression profiles of exhaustion associated genes and *CD5* for each branch.

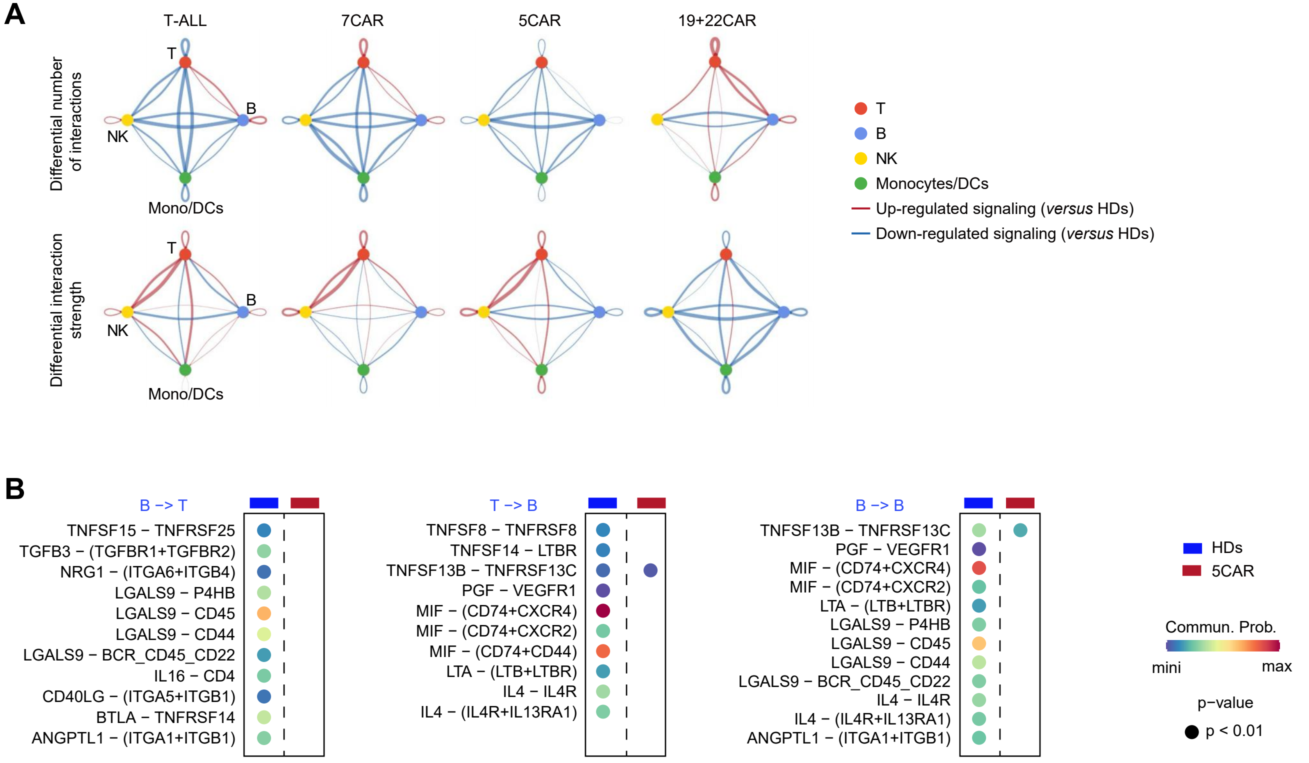
A
T-ALL
7CAR
5CAR
19+22CAR
Differential number of interactions
Differential interaction strength
T
B
NK
Mono/DCs
T
B
NK
Monocytes/DCs
Up-regulated signaling (*versus* HDs)
Down-regulated signaling (*versus* HDs)
B
B -> T
TNFSF15 − TNFRSF25
TGFB3 − (TGFBR1+TGFBR2)
NRG1 − (ITGA6+ITGB4)
LGALS9 − P4HB
LGALS9 − CD45
LGALS9 − CD44
LGALS9 − BCR_CD45_CD22
IL16 − CD4
CD40LG − (ITGA5+ITGB1)
BTLA − TNFRSF14
ANGPTL1 − (ITGA1+ITGB1)
T -> B
TNFSF8 − TNFRSF8
TNFSF14 − LTBR
TNFSF13B − TNFRSF13C
PGF − VEGFR1
MIF − (CD74+CXCR4)
MIF − (CD74+CXCR2)
MIF − (CD74+CD44)
LTA − (LTB+LTBR)
IL4 − IL4R
IL4 − (IL4R+IL13RA1)
B -> B
TNFSF13B − TNFRSF13C
PGF − VEGFR1
MIF − (CD74+CXCR4)
MIF − (CD74+CXCR2)
LTA − (LTB+LTBR)
LGALS9 − P4HB
LGALS9 − CD45
LGALS9 − CD44
LGALS9 − BCR_CD45_CD22
IL4 − IL4R
IL4 − (IL4R+IL13RA1)
ANGPTL1 − (ITGA1+ITGB1)
HDs
5CAR
Commun. Prob.
mini
max
p−value
p < 0.01

**Figure S13. The cell-cell communication networks.**

(**A**) Circle plots showing the different number (top) and strength (bottom) of interactions between any two cell types. The red lines indicate the up-regulated signaling and the blue lines indicate the down-regulated signaling, respectively, compared to HDs.

(**B**) Comparison of the significant ligand-receptor pairs across different groups. Dot color represents the communication probability of the specific ligand-receptor pair between sender cells and receiver cells. *P* value by Cellchat software.

CAR, chimeric antigen receptor; Commun., communication; DCs, dendritic cells; HDs, healthy donors; Mono, monocyte; NK, natural killer cell; Prob., probability; T-ALL, T-cell acute lymphoblastic leukemia.

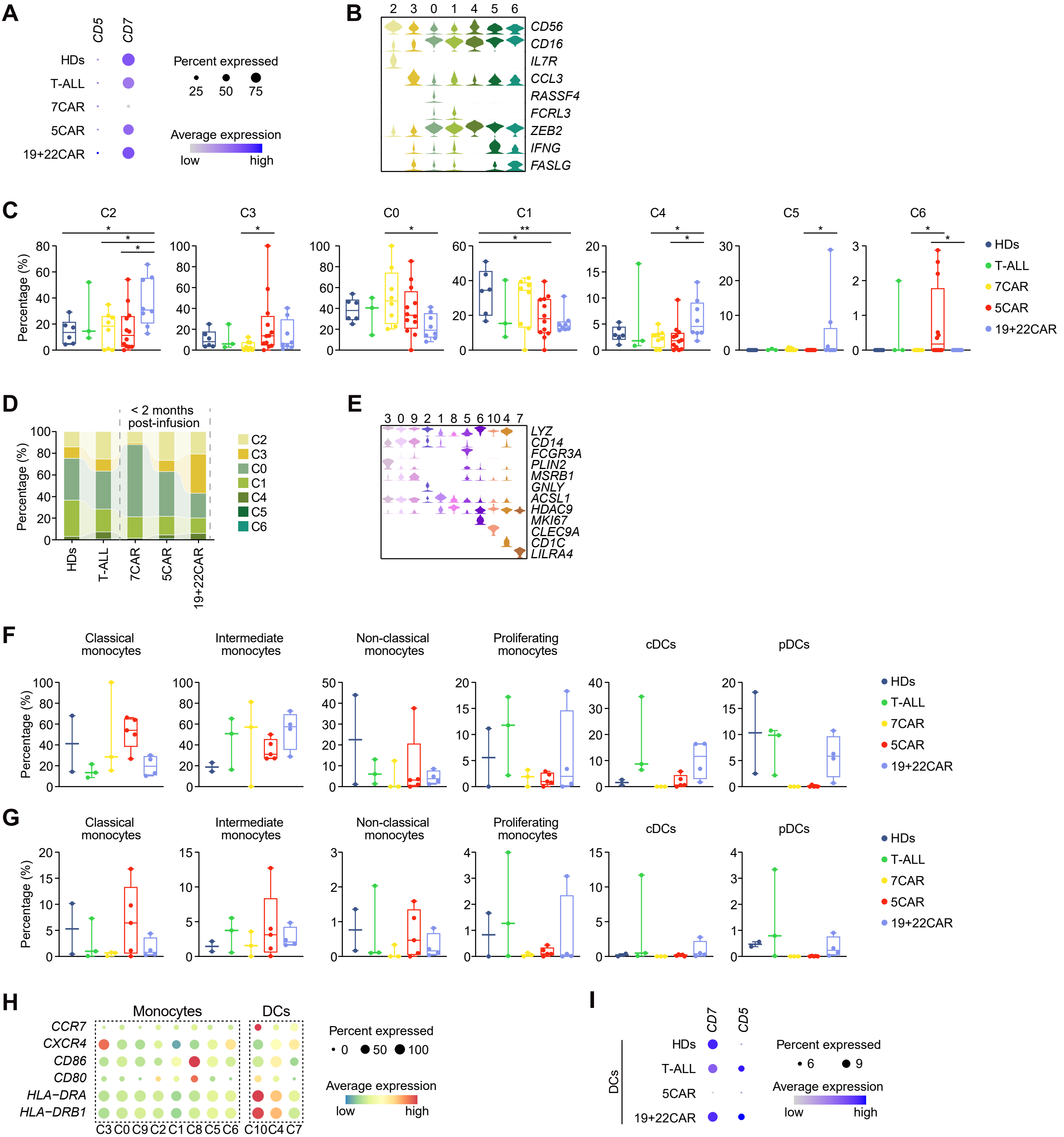

A
CD5
CD7
HDs
T-ALL
7CAR
5CAR
19+22CAR
Percent expressed
25 50 75
Average expression
low high
B
2 3 0 1 4 5 6
CD56
CD16
IL7R
CCL3
RASSF4
FCRL3
ZEB2
IFNG
FASLG
C
C2
C3
C0
C1
C4
C5
C6
Percentage (%)
HDs
T-ALL
7CAR
5CAR
19+22CAR
D
< 2 months post-infusion
Percentage (%)
HDs
T-ALL
7CAR
5CAR
19+22CAR
C2
C3
C0
C1
C4
C5
C6
E
3 0 9 2 1 8 5 6 10 4 7
LYZ
CD14
FCGR3A
PLIN2
MSRB1
GNLY
ACSL1
HDAC9
MKI67
CLEC9A
CD1C
LILRA4
F
Classical monocytes
Intermediate monocytes
Non-classical monocytes
Proliferating monocytes
cDCs
pDCs
Percentage (%)
G
Classical monocytes
Intermediate monocytes
Non-classical monocytes
Proliferating monocytes
cDCs
pDCs
Percentage (%)
H
Monocytes
DCs
CCR7
CXCR4
CD86
CD80
HLA−DRA
HLA−DRB1
C3 C0 C9 C2 C1 C8 C5 C6 C10 C4 C7
Percent expressed
0 50 100
Average expression
low high
I
CD7
CD5
DCs
HDs
T-ALL
5CAR
19+22CAR
Percent expressed
6 9
Average expression
low high

**Figure S14. scRNA-seq reveals heterogeneity of NK, monocytes, and dendritic cell following different CAR T-cell therapies.**

(**A**) Dot plot showing the expression of *CD7* and *CD5* in NK cells in different groups. The dot size represents the percentage of cells expressing the indicated genes, and the dot color shows the averaged expression level.

(**B**) Violin plots showing marker genes in each NK-cell cluster.

(**C**) Comparison of the percentage of specific NK cell populations in different groups across all samples. Boxplots show median ± IQR, whiskers indicate full range, n = 6 in HDs, n = 3 in T-ALL, n = 8 in 7CAR, n = 12 in 5CAR, n = 8 in 19+22CAR group. Dots represent individual samples. *P* value by two-sided unpaired Mann-Whitney test.

(**D**) Relative percentages of NK cell clusters in all healthy donors, CAR-naïve T-ALL patients, and patients within 2 months of CAR T-cell therapies.

(**E**) Violin plots showing marker genes in each monocyte and dendritic cell cluster.

(**F**) Percentages of specific monocyte and dendritic cell populations in all monocytes/DCs in different groups. Boxplots show median ± IQR, whiskers indicate full range, n = 2 in HDs, n = 3 in T-ALL, n = 3 in 7CAR, n = 5 in 5CAR, n = 4 in 19+22CAR group. Dots represent individual samples.

(**G**) Percentages of specific monocyte and dendritic cell populations in all mononuclear cells in different groups. Boxplots show median ± IQR, whiskers indicate full range, n = 2 in HDs, n = 3 in T-ALL, n = 3 in 7CAR, n = 5 in 5CAR, n = 4 in 19+22CAR group. Dots represent individual samples.

(**H**) Dot plot showing the expression of the indicated genes in different subpopulations. The dot size represents the percentage of cells expressing the indicated genes, and the dot color shows the

averaged expression level.

(**I**) Dot plot showing the expression of *CD7* and *CD5* in dendritic cells in different groups. The dot size represents the percentage of cells expressing the indicated genes, and the dot color shows the averaged expression level.

*$p < 0.05$, **$p < 0.01$. CAR, chimeric antigen receptor; cDCs, conventional dendritic cells; DCs, dendritic cells; HDs, healthy donors; pDCs, plasmacytoid dendritic cells; T-ALL, T-cell acute lymphoblastic leukemia.

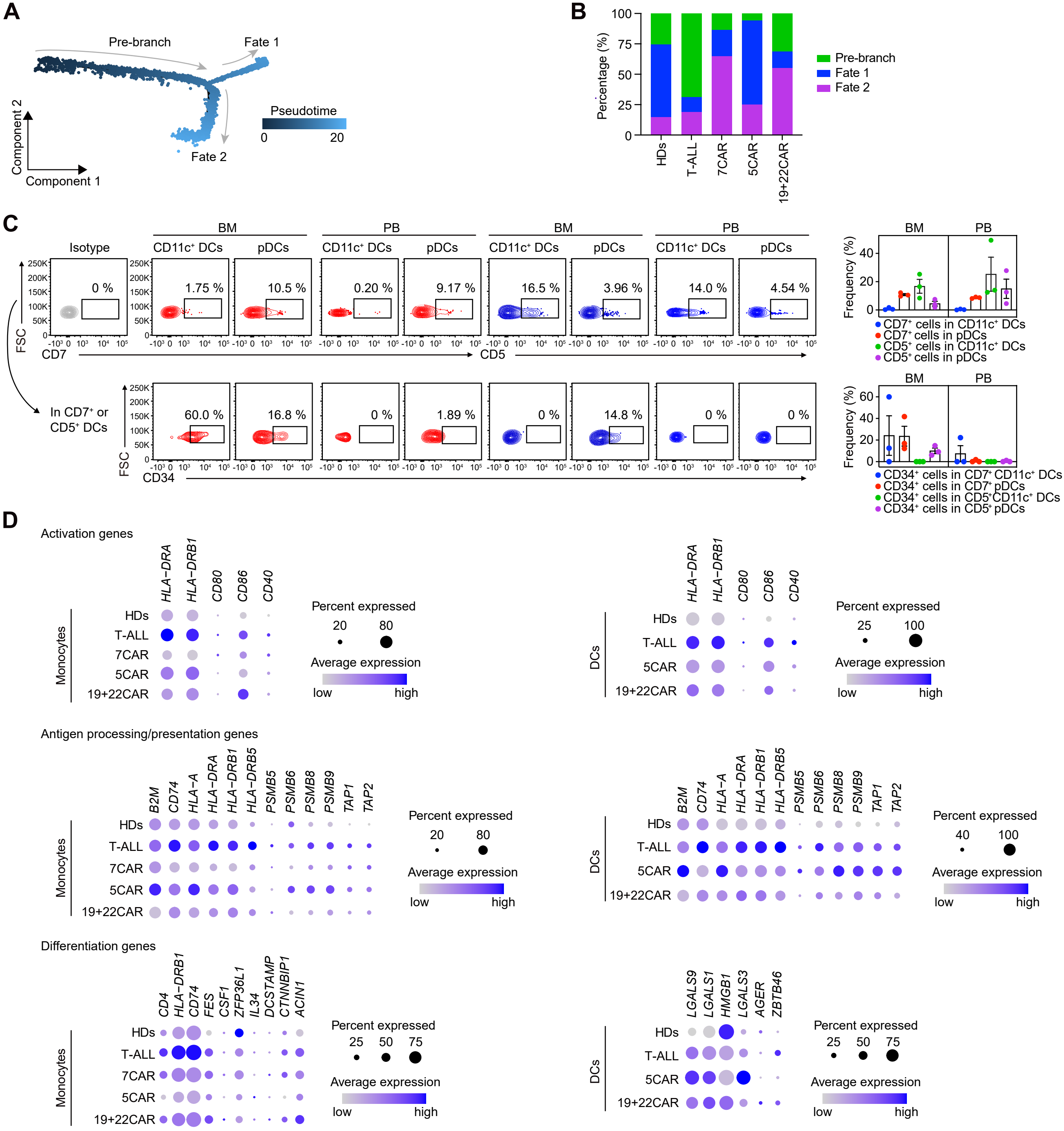
A
Pre-branch
Fate 1
Fate 2
Pseudotime
0
20
Component 1
Component 2
B
Percentage (%)
Pre-branch
Fate 1
Fate 2
HDs
T-ALL
7CAR
5CAR
19+22CAR
C
Isotype
BM
PB
CD11c+ DCs
pDCs
0 %
1.75 %
10.5 %
0.20 %
9.17 %
16.5 %
3.96 %
14.0 %
4.54 %
FSC
CD7
CD5
In CD7+ or CD5+ DCs
60.0 %
16.8 %
0 %
1.89 %
0 %
14.8 %
0 %
0 %
CD34
Frequency (%)
CD7+ cells in CD11c+ DCs
CD7+ cells in pDCs
CD5+ cells in CD11c+ DCs
CD5+ cells in pDCs
CD34+ cells in CD7+ CD11c+ DCs
CD34+ cells in CD7+ pDCs
CD34+ cells in CD5+CD11c+ DCs
CD34+ cells in CD5+ pDCs
D
Activation genes
HLA-DRA
HLA-DRB1
CD80
CD86
CD40
Monocytes
DCs
HDs
T-ALL
7CAR
5CAR
19+22CAR
Percent expressed
Average expression
low
high
Antigen processing/presentation genes
B2M
CD74
HLA-A
HLA-DRA
HLA-DRB1
HLA-DRB5
PSMB5
PSMB6
PSMB8
PSMB9
TAP1
TAP2
Differentiation genes
CD4
HLA-DRB1
CD74
FES
CSF1
ZFP36L1
IL34
DCSTAMP
CTNNBIP1
ACIN1
LGALS9
LGALS1
HMGB1
LGALS3
AGER
ZBTB46

**Figure S15. Pseudotime and gene expression analysis of monocytes and dendritic cells.**

(**A**) Pseudotime trajectory of monocytes and dendritic cells. Dots represent individual cells.

(**B**) The proportions of different monocyte and dendritic cell paths in each group.

(**C**) Expression of CD7, CD5, and CD34 in DCs from HDs measured by flow cytometry. Expression of CD7 and CD5 in $CD11c^+$ DCs or pDCs (left, top). Expression of CD34 in $CD7^+CD11c^+$ DCs, $CD7^+$ pDCs, $CD5^+CD11c^+$ DCs, or $CD5^+$ pDCs (left, bottom). Percentage of $CD7^+$ or $CD5^+$ cells in indicated cells (right, top) and $CD34^+$ cells in indicated cells (right, bottom). Dots represent individual HDs. Mean ± SEM, n = 3 in PB, and n = 3 in BM.

(**D**) Dot plot showing the expression of representative activation, antigen processing/presentation, and differentiation-related genes in monocytes or dendritic cells in different groups. Dot size represents the percentage of cells expressing the indicated genes, and dot color shows the averaged level of expression.

CAR, chimeric antigen receptor; DCs, dendritic cells; HDs, healthy donors; T-ALL, T-cell acute lymphoblastic leukemia.

**Supplementary Tables**

Table S1. Characteristics of healthy donors and patients.

Table S2. Sequencing information of individual samples.

Table S3. Gene signatures of cell clusters.

Table S4. Percentage of clusters in peripheral blood and bone marrow.

Table S5. Gene signatures of T cell clusters.

Table S6. Representative GO pathways.

Table S7. Expression of CD5 on normal T cells, determined by flow cytometry.

Table S8. scTCR-seq metadata.

Table S9. Gene signatures of B cell clusters.

Table S10. scBCR-seq metadata.

Table S11. Gene signatures of NK cell clusters.

Table S12. Gene signatures of monocyte and dendritic cell clusters.

Table S13. List of gene sets related to specific cell features.

## References


1. P. Dash, A. J. Fiore-Gartland, T. Hertz *et al.*, Quantifiable predictive features define epitope-specific T cell receptor repertoires. *Nature* **547**, 89-93 (2017). https://doi.org/10.1038/nature22383

2. R. O. Emerson, W. S. DeWitt, M. Vignali *et al.*, Immunosequencing identifies signatures of cytomegalovirus exposure history and HLA-mediated effects on the T cell repertoire. *Nat Genet* **49**, 659-665 (2017). https://doi.org/10.1038/ng.3822

3. E. H. Akama-Garren, T. van den Broek, L. Simoni *et al.*, Follicular T cells are clonally and transcriptionally distinct in B cell-driven mouse autoimmune disease. *Nat Commun* **12**, 6687 (2021). https://doi.org/10.1038/s41467-021-27035-8

4. M. E. Snyder, K. Moghbeli, A. Bondonese *et al.*, Modulation of tissue resident memory T cells by glucocorticoids after acute cellular rejection in lung transplantation. *J Exp Med* **219**, (2022). https://doi.org/10.1084/jem.20212059

5. S. I. Ramirez, F. Faraji, L. B. Hills *et al.*, Immunological memory diversity in the human upper airway. *Nature* **632**, 630-636 (2024). https://doi.org/10.1038/s41586-024-07748-8
